# Supplementary material for: Superpixel-ComBat multi-site harmonization of unpaired T1W MRI data in Huntington’s disease
Source: Neuroimage Rep. 2026 Nov 4;6(4):100430. doi: 10.1016/j.ynirp.2026.100430 (PMC13753013; doi:10.1016/j.ynirp.2026.100430)
Supplement: Supplementary file 1 [file mmc1.pdf]

Superpixel-ComBat multi-site harmonization of unpaired T1W MRI data in Huntington's disease.

Supplementary materials

Annabelle Coleman[1], Chang-Le Chen[2], Joelle Hanson-Baiden[1], Davneet S Minhas[3], Mahbaneh E Torbati[4], Charles M. Laymon[2,3], Sarah J Tabrizi[1,5], Edward J Wild[1], Dana L Tudorascu[6,7], Rachael I Scahill[1†], Lauren M Byrne[1†]

Author affiliations:

1 Huntington's Disease Centre, Department of Neurodegenerative disease, UCL Queen Square Institute of Neurology, University College London, London, UK

2 Department of Bioengineering, Swanson School of Engineering, University of Pittsburgh, Pittsburgh, PA, USA

3 Department of Radiology, University of Pittsburgh School of Medicine, Pittsburgh, PA, USA

4 Department of Anesthesiology, Perioperative and Pain Medicine, Stanford University, Stanford, CA, USA

5 UK Dementia Research Institute, University College London, London, UK

6 Department of Psychiatry, School of Medicine, University of Pittsburgh, Pittsburgh, PA, USA

7 Department of Biostatistics and Health Data Science, University of Pittsburgh, Pittsburgh, PA, USA

[†] Joint senior authors

Correspondence to: Annabelle Coleman and Dr Lauren M Byrne

Full address: Huntington's Disease Centre, UCL Queen Square Institute of Neurology, 2nd Floor Russell Square House, 10-12 Russell Square, London, WC1B 5EH

Email: annabelle.coleman.21@ucl.ac.uk; lauren.byrne.14@ucl.ac.uk

1 Supplementary methods

1.1 Participant cohorts

Data from three retrospectively collected cohorts were used for harmonization (HD-YAS, HD-CSF, TRACK-HD). HD-YAS is an ongoing single-site longitudinal study investigating the earliest manifestations of HD pathology in pwHD ~24 years from predicted CMD at baseline (Scahill et al., 2020, 2025). In 2018, 67 healthy controls and 64 pwHD underwent clinical and cognitive testing, 3-tesla (3T) volumetric MRI, and biofluid collections (blood and CSF). pwHD required a positive HD genetic test with CAG ≥40, disease burden score (DBS: age x (CAG-35.5); Penney et al. 1997) < 240, and Unified HD Rating Scale (UHDRS) total motor score (TMS; Kieburtz 1996) < 5. Healthy controls were age- and sex-matched gene-negative individuals.

HD-CSF was a single-site two-year longitudinal investigation of CSF in 80 participants (20 healthy controls, 20 pre-CMD, and 40 post-CMD) who had matched 3T T1W MRI and blood collection (Byrne et al., 2018; Rodrigues et al., 2020). Pre-CMD participants had CAG ≥40 with diagnostic confidence score (DCS; Kieburtz 1996) of < 4. Early post-CMD participants had CAG ≥40, DCS = 4, and were staged by UHDRS total functional capacity (TFC; Martinez-Martin et al. 2017) score (Stage 1, TFC 11-13; Stage 2, TFC 10-7; Stage 3, TFC 7-10).

TRACK-HD was a four-year multi-site observational study evaluating biomarkers of disease progression in 366 participants (healthy controls, pre-CMD, and post-CMD) across four sites: London; Leiden; Paris; and Vancouver (Tabrizi et al., 2009, 2011, 2012, 2013). Annual assessments included 3T T1W MRI, blood sampling, and clinical, motor, and cognitive assessments. All pwHD had a CAG ≥40. Pre-CMD participants required DBS >250 and a TMS ≤5, indicating no substantial clinical motor signs. Post-CMD participants had a DCS of 4, and were within Shoulson and Fahn stage 1 or 2 (TFC ≥7; Shoulson and Fahn 1979). Healthy controls were age- and sex-matched spouses, partners, or gene negative siblings.

Detailed acquisition protocols for each dataset have been previously published (Byrne et al., 2018; Scahill et al., 2020; Tabrizi et al., 2009).

1.2 Criteria for pipeline selection

The accumulation of results from each pipeline were evaluated and a decision was made about which pipeline to choose for larger-scale analysis (Supplementary Table 7). The aim was to identify a pipeline that most effectively mitigated scanner-related bias while preserving biologically meaningful variability and anatomical integrity. Each pipeline was evaluated across four key domains: (1) scanner bias reduction; (2) biological variability preservation; (3) structural integrity; (4) computational feasibility. While the ideal design would excel across all evaluation domains, trade-offs may be necessary. In cases

where designs perform similarly, preference was given to feasible or more computationally efficient options. If none of the evaluated experimental designs met an acceptable standard due to persistent distortions or inadequate scanner effect removal, a standard unharmonized pipeline would be used with scanner as a covariate (no harmonization procedures).

1.3 VBM statistical analysis contrast design

Voxel-wise comparisons on both grey and white matter between unharmonized and harmonized TPMs were conducted to identify any systematic changes in brain morphology introduced by SP-ComBat. We anticipated this whole-brain unbiased voxel-wise approach would provide information on regional biases; uncover specific brain regions that were more impacted by harmonisation due to scanner effects or disease-specific morphological differences. All voxel-wise analyses were conducted using SPM12.

Flexible factorial design specification

For within scanner and within disease comparisons, a general linear model (GLM) with a flexible factorial design questioned if harmonization impacted the resulting grey and white matter values, and whether any influence was dependent on a specific scanner of disease group. Two separate GLMs were constructed: one for grey matter TPMs and one for white matter TPMs. The design matrix included two predictors (Supplementary Figure 2A-B): (1) harmonization (two levels: unharmonized and harmonized) and (2) scanner (six levels: HD-YAS, HD-CSF, TRACK-HD London, TRACK-HD Leiden, TRACK-HD Paris, and TRACK-HD Vancouver) or disease group (three levels: controls, pre-CMD, and post-CMD). Covariates included age, sex, and TIV.

ANOVA design specification

To assess disease group differences in the unharmonized and harmonized datasets, independent voxel-wise one-way ANOVAs were built to question if there were significant differences between grey/white matter regions between each disease group. Four different one-way ANOVAs were performed: unharmonised grey (1) and white (2) matter TPMs, harmonized grey (3) and white (4) matter TPMs. The design matrix included one predictor (Supplementary Figure 2C): disease group (three levels: controls, pre-CMD, and post-CMD). Covariates included age, sex, and TIV. Scanner was added as a covariate in the unharmonized ANOVA.

Contrast specification

GLMs were estimated using the 'Estimate' batch job in SPM12. To examine scanner- and disease-specific harmonisation effects or disease group differences, the ‘Results’ batch job was used to define F- and T-contrasts (Supplementary Table 8). For the effects of harmonization, F-contrasts were specified to identify regions where harmonisation significantly altered grey/white matter values across all scanners or disease groups, while T-contrasts were used to detect scanner- or disease- specific

increases or decreases in grey/white matter values due to harmonisation. For disease group differences comparing pwHD to healthy controls, T-contrasts were specified to identify regions with significantly different grey/white matter values between healthy controls and pre-CMD participants, and healthy controls and post-CMD participants.

2 Supplementary results

2.1 Effect of bootstrap resampling on scanner-effect estimation

To clarify how bootstrap resampling affects SP-ComBat scanner-effect estimation, we conducted two post-hoc analyses examining the contribution of resampling itself. The first held pseudo-pair sample size constant, isolating the contribution of resampling itself; the second examined resampling's effect in an independent cohort across a range of pool sizes to test whether the deficit was explained by sample size alone.

First, within the present dataset, we constructed a bootstrapped variant of Pipeline 2 (Pipeline 3) by resampling its 16 directly matched pseudo-pairs with replacement to generate 25 synthetic pairs, matching the resampling scheme applied to Pipeline 1's four pairs. Because Pipeline 3 and Pipeline 2 were estimated from an identical set of sixteen pseudo-pairs and differed only in whether those pairs were bootstrapped, comparable re-estimated parameter maps would be expected if pseudo-pair number, rather than resampling, governed estimation stability. Instead, Pipeline 3's re-estimated additive (γ) and multiplicative (δ) maps showed markedly greater residual scanner-related structure than Pipeline 2's un-resampled maps, most pronounced for HD-CSF (Supplementary Figure 3). This suggests that the instability observed in Pipeline 1 reflects bootstrap resampling itself rather than its smaller base pseudo-pair pool.

Second, to test whether this resampling-driven degradation generalizes beyond the present dataset and pseudo-pairing framework, we performed an independent convergence experiment using the derived site-effect parameters from the traveling-subject dataset underlying the original SP-ComBat validation study (Chen et al., 2024). Unlike the present study's pseudo-paired surrogate controls, this dataset comprised genuine traveling subjects scanned across four scanner platforms (GE, Siemens Prisma, Siemens Trio, and Philips; N = 19, except the Phillips scanner, where N = 15), independent of the HD-YAS, HD-CSF, and TRACK-HD scanners used here. This analysis tested how closely scanner-specific parametric maps matched a known ground truth as sample size increased. Two aggregation methods were compared: direct averaging of unique subjects ("regular permutation") and bootstrap resampling with replacement to a bootstrapped pool of 19. Ground-truth additive and multiplicative parametric maps for each scanner were defined as those estimated from the full available sample (N = 19, except one scanner). Similarity to this ground truth at each pool size was quantified using the structural similarity index measure (SSIM). Regular permutation showed a strong, near-monotonic improvement in map similarity with increasing pool size, approaching an SSIM of 1.0 at the full available sample for each scanner. Bootstrapping also improved with pool size but consistently underperformed regular permutation, plateaued well below the ground truth even when the pool size matched the full available dataset, and showed persistently wider variability across repetitions (Supplementary Figure 4). Because this deficit persisted even at maximum sample size, it cannot be attributed to an insufficient pool of

subjects, indicating that resampling itself, rather than pseudo-pair number, is the limiting factor. This pattern was consistent across all four scanner platforms and both parameters.

Together, these two independent analyses indicate that the reduced stability of Pipeline 1 was driven specifically by bootstrap resampling rather than pseudo-pair sample size, and that this effect is not specific to the present dataset or harmonization pipeline.

2.2 T1W MRI QC

All pseudo-paired control T1W MRI scored 0 on the QC severity scale with no artifacts detected.

For the RMD, 127/144 (88%) scans scored 0 on the severity scale with no artifacts detected, 12/144 (8%) scored 1 with minor artefacts with half being from HD-CSF scanner, and 5/144 (3%) scored 2 with mild artefacts that were slightly more prominent. 4/5 of the scans that were rated a score of 2 were from the post-CMD HD group. Although some artifacts persisted, none were deemed bad enough for exclusion. After harmonization, for both pipelines the visual QC outcomes were identical to those of the unharmonized T1W MRI.

For the FMD, 469/548 (86%) scans scored 0 on the QC severity scale with no artifacts detected, 41/548 (7%) scored 1 with minor artefacts, 23/548 (4%) scored 2 with mild artefacts that were slightly more prominent, 5/548 (1%) scored 3 with moderate artefacts, and 7/548 (1%) scored 4 and above with heavy artefacts detected (excluded;

Supplementary Table 9). All scans that were rated 3 or higher were pwHD post-CMD. Three scans were excluded due to the presence of non-HD pathology. This gave an exclusion rate of 1.8% in the raw T1W QC, leaving 538 scans for harmonization. After harmonization, the same QC results were replicated as before harmonization with all n = 538 passing visual QC.

2.3 Sample size consistency

Sample sizes differ across analyses due to analysis-specific QC criteria applied at each stage. The RMD comprised 144 participants with available T1W MRI; none scored above the exclusion threshold for raw T1W visual QC (> 3 on visual QC severity rating scale; Supplementary Table 2), so all 144 were retained for pipeline development and comparison, with no further exclusions applied at the TPM segmentation QC stage.

The FMD comprised 548 participants with available T1W MRI (Supplementary Figure 8); following raw T1W visual QC, scans scoring above 3 on the visual QC severity rating scale (Supplementary Table 2) or showing non-HD pathology were excluded, leaving 538 scans for image-level quality, inter-scanner variability analyses, and downstream VBM pre-processing. A further criterion was then applied at the level of TPM segmentation QC (>3 on the TPM QC severity rating scale; Supplementary Table 6). Where either the grey or white matter TPM failed QC for a given participant, both TPMs were

excluded for that participant, across both unharmonized and harmonized statuses. Of the 538 FMD participants, 37 were excluded due to non-systematic segmentation errors (sporadic tissue misclassifications unrelated to scanner identity, most commonly meningeal over-segmentation) and a further 122 were excluded due to systematic segmentation errors (consistent, scanner-driven misclassification affecting HD-YAS and HD-CSF scans, comprising grey matter over-segmentation and white matter under-segmentation; Supplementary Table 15; Supplementary Figure 8). For scanner-specific voxel-wise and volumetric analyses, only TPMs with non-systematic errors were excluded; systematic-error scans were retained to characterize the extent of these scanner-driven segmentation failures and their resolution by harmonization, leaving 501 matched unharmonized/harmonized TPMs. For disease-group-specific voxel-wise and volumetric analyses, TPMs with either systematic or non-systematic errors were excluded, so that observed disease-group differences reflected true biological variation rather than residual scanner-driven segmentation error, yielding the final analysis sample of 379 participants.

3 Supplementary Tables

Supplementary Table 1: MRI acquisition parameters for each scanner across the three study cohorts

Study	HD-YAS	HD-CSF	TRACK-HD London	TRACK-HD Leiden	TRACK-HD Paris	TRACK-HD Vancouver
**Location**	London	London	London	Leiden	Paris	Vancouver
**Scanner**	Siemens Prisma	Siemens Prisma	Siemens Trio	Philips Achieva	Siemens Trio	Philips Achieva
**Field (T)**	3	3	3	3	3	3
**Sequence**	T1W 3D MPRAGE	T1W 3D MPRAGE	T1W 3D MPRAGE	T1W 3D MPRAGE	T1W 3D MPRAGE	T1W 3D MPRAGE
**Plane**	Sagittal	Coronal	Sagittal	Sagittal	Sagittal	Sagittal
**Voxel size (mm^3)**	1x1x1	1x1x1	1x1x1	1x1x1	1x1x1	1x1x1
**ST (mm)**	1	1	1	1	1	1
**TR (ms)**	2530	2000	2200	7.7	2200	7.7
**TE (ms)**	3.34	2.05	2.2	3.5	2.2	3.5
**TI (ms)**	1100	850	900	950	900	950
**FA (°)**	7	8	10	8	10	8
**FOV (mm^3)**	256	240	280	240	280	240
**Matrix size**	224x224	256x240	256x256	224x224	256x256	224x224

Scanner specific acquisition details outlined in retrospective studies (Byrne et al., 2018; Scahill et al., 2020; Tabrizi et al., 2009).
FA, flip angle; FOV, field of view; HD, Huntington's disease; HD-CSF; HD Cerebrospinal Fluid Study; HD-YAS, HD Young Adult Study; MPRAGE, Magnetization Prepared Rapid Gradient Echo; ST, slice thickness; T, tesla; T1W, T1 weighted; TE, echo time; TI, inversion time; TR, repetition time.

Supplementary Table 2: Visual quality control severity rating scale for raw T1-weighted MRI

Severity	Rating	Artefact density	Distribution through scan	Image disruption
**None**	**0**	No artefacts detected	Throughout the image	None
**Mild**	**1**	Minor artefacts	On many slices throughout the image	Small distortion of anatomical structures
	**2**	Slightly more prominent	Restricted to one or two slices	
**Moderate**	**3**	Slightly more prominent	On many slices throughout the image	Slight distortion of anatomical structures
**Severe**	**4**	Heavy artefacts	Restricted to one or two slices	Significantly distort anatomical structures
	**5**	Heavy artefacts	Throughout the image in both hemispheres, distorting anatomical structures	

Raw T1W MRI with scores >3 excluded. QC, quality control; T1W, T1-weighted.

Supplementary Table 3: Pseudo-pair demographic information for both pipelines

Pseudo-pair	Sex	Age	
		**Mean (SD)**	**Range**
**Pipeline 1**			
1	F	39.27 (2.13)	38.1 – 43.6
2	F	44.95 (0.41)	44.2 – 45.4
3	M	38.57 (1.16)	37.2 – 39.8
4	M	40.93 (1.06	39.6 – 42.7
**Pipeline 2**			
1	F	34.72 (2.52)	30.8 – 38.4
2	F	37.65 (3.21)	35.2 – 43.6
3	F	39.67 (2.69)	38.1 – 45.1
4	F	41.58 (2.14)	39.8 – 42.5
5	F	44.63 (1.59)	41.8 – 46.6
6	F	45.93 (1.89)	43.5 – 49.1
7	F	49.92 (2.87)	44.2 – 51.9
8	F	52.08 (3.67)	44.9 – 55.1
9	M	35.87 (1.93)	33.8 – 39.5
10	M	37.52 (1.36)	35.4 – 39.5
11	M	38.33 (1.14)	37.0 – 39.9
12	M	40.50 (1.17)	39.6 – 42.7
13	M	43.65 (3.85)	40.4 – 50.7
14	M	45.67 (3.68)	41.8 – 52.6
15	M	47.17 (3.70)	43.0 – 54.1
16	M	48.72 (3.35)	43.8 – 54.2

Age reported as mean (SD) and range across all scanners within each pseudo-pair. Each pseudo-pair consisted of one healthy control per scanner matched on age and sex. F, female; M, male; SD, standard deviation.

Supplementary Table 4: RMD participants used per scanner for pipeline optimization and harmonization assessment

Study	Healthy controls (n)	pre-CMD (n)	post-CMD (n)	Total (n)
**HD-YAS**	16	16	-	32
**HD-CSF**	16	16	20	52
**TRACK-HD London**	4	4	7	15
**TRACK-HD Leiden**	4	4	7	15
**TRACK-HD Paris**	4	4	7	15
**TRACK-HD Vancouver**	4	4	7	15
**Total**	**48**	**48**	**48**	**144**

Each TRACK-HD site operated an independent scanner; HD-YAS and HD-CSF were each acquired on a single scanner. HD, Huntington's disease; HD-CSF, HD Cerebrospinal Fluid Study; HD-YAS, HD Young Adult Study; post-CMD, people with HD after clinical motor diagnosis; pre-CMD, people with HD before clinical motor diagnosis.

Supplementary Table 5: FMD participants for scaled-up harmonization

Study	Healthy controls (n)	pre-CMD (n)	post-CMD (n)	Total (n)
**HD-YAS**	61	62	-	123
**HD-CSF**	16	16	33	65
**TRACK-HD London**	29	30	30	89
**TRACK-HD Leiden**	30	30	30	90
**TRACK-HD Paris**	30	30	26	86
**TRACK-HD Vancouver**	33	30	32	95
**Total**	**198**	**198**	**151**	**548**

Each TRACK-HD site operated an independent scanner; HD-YAS and HD-CSF were each acquired on a single scanner. HD, Huntington's disease; HD-CSF, HD Cerebrospinal Fluid Study; HD-YAS, HD Young Adult Study; post-CMD, people with HD after clinical motor diagnosis; pre-CMD, people with HD before clinical motor diagnosis.

Supplementary Table 6: Visual quality control severity rating scale for grey and white matter tissue probability maps

<table>
<tr><th>Severity</th><th>Rating</th><th>Artefact density</th><th>Distribution through scan</th><th>Image disruption</th></tr>
<tr><td>0</td><td>None</td><td>No segmentation errors detected</td><td>Throughout the image</td><td>None</td></tr>
<tr><td>1</td><td rowspan="2">Mild</td><td>Minor misclassifications or boundary errors</td><td>On many slices throughout the image</td><td rowspan="2">Small tissue class misclassification</td></tr>
<tr><td>2</td><td>Slightly more prominent</td><td>Restricted to one or two slices</td></tr>
<tr><td>3</td><td>Moderate</td><td>Slightly noticeable boundary inaccuracies or tissue misclassification</td><td>On many slices throughout the image</td><td>Slight incorrect tissue classification</td></tr>
<tr><td>4</td><td rowspan="2">Severe</td><td>Heavy artefacts</td><td>Restricted to one or two slices</td><td rowspan="2">Significantly distort anatomical structures</td></tr>
<tr><td>5</td><td>Heavy artefacts</td><td>Throughout the image in both hemispheres, distorting anatomical structures</td></tr>
</table>

TPMs with scores >3 excluded. If the TPM for one tissue class was excluded, all corresponding TPMs for that participant were excluded, both unharmonized and harmonized. QC, quality control; TPM, tissue probability map.

Supplementary Table 7: Criteria for go/no-go decisions for pipeline selection

Category	Assessment method from each pipeline	Outcome measures to compare between pipelines	Deciding factors to choose a pipeline for FMD
		**Image quality assessments**	
**Visual QC**	T1W MRI visual QC	Artifact rating scale (0-5).	Low average artifact rating, with a minimal number of scans rated > 3.
**Segmentation consistency**	Grey/white matter TPM overlay	Visual comparison of segmentation overlays.	Minimal significant distortion of anatomical boundaries and may even demonstrate improved tissue assignment.
**Image quality metrics**	MRIQC (SNR, CNR, CJV)	SNR, CNR, and CJV statistical differences.	SNR and CNR values that are stable or higher than the comparative pipeline and CJV that are stable or lower.
**Voxel contrast intensity distributions**	Intensity plots	Plots of intensity distributions from different scanners.	More aligned intensity distributions from different scanners while preserving biologically meaningful variation (tissue-class and disease-related differences).
		**Voxel-wise assessments**	
**Scanner-wise assessment**	Paired voxel-wise analysis (flexible factorial GLM)	Peak T-values; number of significant voxels/clusters; spatial location of clusters.	Fewer/smaller scanner-driven clusters, lower peak T-values, minimal overlap with biologically important ROIs.
**Within disease groups**	Paired voxel-wise analysis (flexible factorial GLM)	Peak T-values; number of significant voxels/clusters; spatial location of clusters.	Fewer/smaller within-group clusters, lower peak T-values, minimal overlap with biologically important ROIs.
**Disease group preservation**	Independent voxel-wise analysis (ANOVA)	Peak T-values; number of significant voxels/clusters; spatial location of clusters.	Disease-related clusters, higher peak T-values, spatial location within expected disease-relevant ROIs.
		**Volumetric assessments**	
**Overall volume changes**	Linear mixed-effects modelling of volumes from TPMs	Harmonization main effect (β, 95% CI, p-value). Post-hoc pairwise contrasts (Bonferroni-adjusted p-values). Mean differences (harmonized − unharmonized).	Minimal systematic bias (small % bias), non-significant or very small harmonization effect size.
**Scanner/disease-specific biases**	Linear mixed-effects modelling of volumes from TPMs	Interaction effects (harmonization × scanner; harmonization × disease: β, 95% CI, p-value). Scanner-/disease-wise post-hoc contrasts (Bonferroni-adjusted p-values). Mean differences by scanner/disease group.	Reduced scanner-driven differences (small/non-significant interactions, small effect sizes), no new scanner × disease interactions, and mean differences within acceptable thresholds.
**Agreement across pipelines**	ICC, spaghetti plots, Bland-Altman plots	ICC values (0–1), visual inspection of spaghetti and Bland-Altman plots for systematic bias.	High ICC (≥0.75 indicating good agreement); spaghetti/Bland-Altman plots showing minimal systematic bias in volumes after harmonization.
		**Advanced assessments**	
**Principal Component Analysis (PCA)**	PCA on Voxel Intensities	Variance explained by top components; clustering by scanner/disease	Less scanner-driven clustering, lower variance explained by scanner-related PCs
**SP-ComBat re-estimation**	Re-estimation of parametric maps	Gamma and delta intensity maps	Additive terms close to zero and multiplicative terms close to one, indicating minimal scanner-driven deviation and alignment to a unified standard distribution.
**CNN classifiers**	CNN for scanner prediction (FMD only)	Scanner prediction accuracy	Lower accuracy (closer to chance), meaning stronger harmonization

ANOVA, analysis of variance; CJV, coefficient of joint variation; CNN, convolutional neural network; CNR, contrast to noise ratio; FMD, full multi-study dataset; GLM, general linear model; MRI, magnetic resonance imaging; PCA, Principal Component Analysis; PC, Principal Component; ROI, region of interest; SNR, signal to noise ratio; SP, superpixel; TPM, tissue probability map; QC, quality control.

Supplementary Table 8: Specification of F- and T-contrast vectors for voxel-wise harmonization and disease group analyses in grey and white matter

Comparison	Contrast	Vector
	Within scanner-specific contrasts	
Main effect of harmonization	F-contrast	[1 -1 1 -1 1 -1 1 -1 1 -1 1 -1]
Harmonized > Unharmonized across all scanners	T-contrast	[-1 1 -1 1 -1 1 -1 1 -1 1 -1 1]
Harmonized > Unharmonized HD-YAS	T-contrast	[0 0 0 0 -1 0 0 0 0 0 1 0]
Harmonized > Unharmonized HD-CSF	T-contrast	[0 0 0 0 0 -1 0 0 0 0 0 1]
Harmonized > Unharmonized THD Paris	T-contrast	[-1 0 0 0 0 0 1 0 0 0 0 0]
Harmonized > Unharmonized THD London	T-contrast	[0 -1 0 0 0 0 0 1 0 0 0 0]
Harmonized > Unharmonized THD Leiden	T-contrast	[0 0 -1 0 0 0 0 0 1 0 0 0]
Harmonized > Unharmonized THD Vancouver	T-contrast	[0 0 0 -1 0 0 0 0 0 1 0 0]
	Within disease group specific contrasts	
Main effect of harmonization	F-contrast	[1 -1 1 -1 1 -1]
Harmonized > Unharmonized across all disease groups	T-contrast	[-1 1 -1 1 -1 1]
Harmonized > Unharmonized HC	T-contrast	[0 -1 0 0 1 0]
Harmonized > Unharmonized pre-CMD	T-contrast	[-1 0 0 1 0 0]
Harmonized > Unharmonized post-CMD	T-contrast	[0 0 -1 0 0 1]
	Between disease group specific contrasts	
HCs > pre-CMD	T-contrast	[1 -1 0]
HCs > post-CMD	T-contrast	[1 0 -1]

[1] represents unharmonized and [-1] represents harmonized. [0] was used to block comparisons in the relevant scanners or disease groups. HC, healthy control; HD, Huntington's disease; HD-CSF, HD Cerebrospinal Fluid Study; HD-YAS, HD Young Adult Study; post-CMD, people with HD after clinical motor diagnosis; pre-CMD, people with HD before clinical motor diagnosis; THD, TRACK-HD study site.

Supplementary Table 9: Raw T1W MRI quality control exclusions by scanner in the FMD

Scanner	Score 0 - 3 (n)	Score 4 - 5, excluded, n (%)	Total
HD-YAS	123	0 (0%)	123
HD-CSF	58	6 (9.4%)	64
THD London	89	0 (0%)	89
THD Leiden	88	2 (2.2%)	90
THD Paris	86	1 (1.1%)	87
THD Vancouver	94	1 (1.1%)	95
Total	538	10 (1.8%)	548

Number of scans retained (severity score 0–3) and excluded at raw T1W visual quality control, by scanner, with the exclusion rate expressed as a percentage of each scanner's total scans. Severity ratings followed the scale in Supplementary Table 2. Excluded scans > 3 on the severity scale, together with three additional scans excluded for evidence of non-HD pathology. HD, Huntington's disease; HD-CSF, HD Cerebrospinal Fluid Study; HD-YAS, HD Young Adult Study; QC, quality control; T1W, T1-weighted; THD, TRACK-HD study site.

Supplementary Table 10: RMD group demographics (n = 144)

Study	Disease group (n)	Age		Sex		CAG		DBS	
		mean (SD), range	p	%M	p	mean (SD), range	p	mean (SD), range	p
**HD-YAS**	Control (16)	36.5 (2.1), 34 - 41.5	0.145[1]	56	1.000	-	-	-	-
	pre-CMD (16)	35.4 (2), 32 - 40		56		41.2 (0.9), 40 - 43		200 (27.6), 153 - 240	
	Control (16)	50.4 (11), 29 - 74		56		-		-	
**HD-CSF**	pre-CMD (16)	41.5 (11.7), 25 - 67	**0.026**[1], 0.194[2], **< 0.005**[3]	56	0.9078	42.3 (1.7), 40 - 45	0.657	272.2 (66.3), 113 - 383	**< 0.005**
	post-CMD (20)	54.6 (8.8), 40.9 - 71.5		50		42.5 (1.7), 40 - 45		370 (72.7), 248 - 493	
	Control (4)	50.8 (8.9), 42.6 - 63.5		50		-		-	
**TRACK-HD London**	pre-CMD (4)	36.8 (3.7), 33.6 - 41	**0.033**[1], 0.810[2], **0.012**[3]	0	0.1580	42.8 (1.5), 41 - 44	0.648	263.3 (34.5), 213 - 287	**0.015**
	post-CMD (7)	52.1 (9.4), 35.8 - 63.8		43		42.3 (1.6), 40 - 45		342.3 (45.1), 287 - 406	
	Control (4)	52.3 (5.3), 47.3 - 58.2		50		-		-	
**TRACK-HD Leiden**	pre-CMD (4)	36.2 (0.7), 35.4 - 36.9	**0.017**[1], 0.97[2], **0.008**[3]	25	0.7059	43.8 (0.96), 43 - 45	0.320	298 (30.8), 270 - 336	**0.008**
	post-CMD (7)	52.5 (11), 31.7 - 63.9		29		42.7 (1.8), 41 - 46		362.2 (29.7), 321 - 398	
	Control (4)	48 (8.2), 38.6 - 58.5		50		-		-	
**TRACK-HD Paris**	pre-CMD (4)	35.3 (2.7), 32.4 - 37.8	**0.031**[1], 0.343[2], **0.002**[3]	25	0.7537	43 (1.4), 42 - 45	0.655	263.2 (39.1), 218 - 308	**0.002**
	post-CMD (7)	52.6 (8.4), 37.4 - 60.9		43		42.6 (1.5), 42 - 46		363 (35.3), 313 - 396	
	Control (4)	51.4 (5.5), 45.6 –58.1		50		-		-	
**TRACK-HD Vancouver**	pre-CMD (4)	36.2 (8.4), 27.5 - 47.3	**0.029**[1], 0.895[2], **0.012**[3]	75	0.7537	43 (1.4), 41 - 44	0.902	262.9 (21.4), 234 - 280	**< 0.005**
	post-CMD (7)	52.1 (9.9), 31.1 - 60.2		57		42.9 (2), 41 - 47		367 (21.8), 331 - 391	

Data expressed as mean (SD), range. Disease groups were compared using Independent One-Way ANOVA (Age, CAG and DBS) and Chi-squared test (Sex). For age, post-estimation Wald tests were used to assess intergroup differences with Bonferroni correction for multiple comparisons. Bold values represent significant comparisons. [1]HC vs pre-CMD, [2]HC vs post-CMD, and [3]pre-CMD vs post-CMD. CAG, cytosine-adenine-guanine; DBS, disease burden score; HC, healthy control; HD, Huntington's disease; HD-CSF, HD Cerebrospinal Fluid Study; HD-YAS, HD Young Adult Study; M, male; SD, standard deviation; post-CMD, pwHD after clinical motor diagnosis; pre-CMD, pwHD before clinical motor diagnosis.

Supplementary Table 11: FMD group demographics after raw scan T1W QC (n= 538)

Study	Disease group (n)	Age		Sex		CAG		DBS	
		mean (SD), range	p	%M	p	mean (SD), range	p	mean (SD), range	p
**HD-YAS**	Control (61)	29.7 (5.5), 20.2 – 40	0.941[1]	39	0.4054	-	-	-	-
	pre-CMD (62)	29.6 (5.7), 19.3 – 40.9		47		42.3 (1.7), 39 - 47		196.2 (37.2), 122 - 316	
**HD-CSF**	Control (15)	49.5 (11), 30 – 74.7	**0.050[1]**, 0.116[2], < **0.005[3]**	53	0.8672	-	0.474	-	**< 0.005**
	pre-CMD (16)	42 (11.7), 26 – 67.5		56		42.3 (1.7), 40 - 45		284 (68.2), 122 – 398	
	post-CMD (27)	54.8 (9.2), 40.7 – 71.5		48		42.7 (1.9), 39 - 45		396.8 (103), 191 - 622	
**TRACK-HD London**	Control (29)	45.1 (9.2), 24 – 63.5	**0.069[1]**, 0.150[2], **0.001[3]**	45	0.8657	-	0.478	-	**< 0.005**
	pre-CMD (30)	40.8 (7.5), 26.1 – 58.5		47		43.1 (1.9), 40 – 48		305.8 (43.7), 221 – 386	
	post-CMD (30)	48.4 (9.8), 29.6 – 64.1		40		43.5 (2.4), 39 - 48		378.4 (79.5), 232 - 541	
**TRACK-HD Leiden**	Control (30)	49.2 (8.3), 35.2 – 65.7	**0.023[1]**, 0.821[2], **0.044[3]**	47	0.5283	-	0.158	-	**< 0.005**
	pre-CMD (30)	43.8 (8), 26.4 – 61.7		40		42.5 (2.4), 39 – 50		301.4 (53.7), 181 - 399	
	post-CMD (28)	48.6 (10.5), 30.7 – 63.9		32		43.4 (2.5), 40 - 50		374.1 (56.4), 284 - 490	
**TRACK-HD Paris**	Control (29)	44.4 (11.2), 25.9 – 63	**0.036[1]**, 0.064[2], < **0.005[3]**	48	0.6346	-	0.299	-	**0.001**
	pre-CMD (30)	39.1 (9.1), 22.3 – 64.1		53		43.7 (2.7) 39 - 52		308 (49.7), 207 - 415	
	post-CMD (27)	49.3 (8.4), 33.7 – 60.9		41		43 (2), 39 - 47		368.8 (74.4), 165 - 507	
**TRACK-HD Vancouver**	Control (33)	45.7 (12), 23 – 62.6	**0.035[1]**, 0.371[2], **0.004[3]**	39	**0.007**	-	0.135	-	**< 0.005**
	pre-CMD (30)	39.6 (10.3), 18.6 – 61.8		40		43.3 (2.5), 40 - 50		296.8 (43.1), 219 - 373	
	post-CMD (31)	48.2 (11.3), 22.8 – 63.4		74		44.7 (4.2), 40 - 59		412.3 (75.2), 280 - 574	

Data expressed as mean (SD), range. Disease groups were compared using Independent One-Way ANOVA (Age, CAG and DBS) and Chi-squared test (Sex). For age, post-estimation Wald tests were used to assess intergroup differences with Bonferroni correction for multiple comparisons. Bold values represent significant comparisons. [1]HC vs pre-CMD, [2]HC vs post-CMD and [3]pre-CMD vs post-CMD. CAG, cytosine-adenine-guanine; DBS, disease burden score; HC, healthy control; HD, Huntington's disease; HD-CSF, HD Cerebrospinal Fluid Study; HD-YAS, HD Young Adult Study; M, male; SD, standard deviation; post-CMD, pwHD after clinical motor diagnosis; pre-CMD, pwHD before clinical motor diagnosis.

Supplementary Table 12: MRIQC image quality metric differences between unharmonized and harmonized T1W MRI

	**Adjusted MD**	**SE**	**95 % CI**	**p-value**
		**CNR**		
Overall	-0.018	0.037	-0.090 to 0.055	0.632
HD-YAS	0.681	0.077	0.531 to 0.832	**< 0.0001**
HD-CSF	0.247	0.113	0.027 to 0.468	**0.028**
THD London	-0.052	0.091	-0.230 to 0.127	0.570
THD Leiden	-0.505	0.091	-0.684 to -0.326	**< 0.0001**
THD Paris	0.008	0.092	-0.173 to 0.190	0.929
THD Vancouver	-0.639	0.088	-0.813 to -0.466	**< 0.0001**
		**CJV**		
Overall	-0.084	0.004	-0.092 to -0.077	**< 0.0001**
HD-YAS	-0.181	0.008	-0.197 to -0.166	**< 0.0001**
HD-CSF	-0.119	0.012	-0.141 to -0.096	**< 0.0001**
THD London	-0.058	0.009	-0.077 to -0.040	**< 0.0001**
THD Leiden	-0.016	0.009	-0.034 to 0.003	0.098
THD Paris	-0.075	0.010	-0.094 to -0.057	**< 0.0001**
THD Vancouver	-0.033	0.009	-0.051 to -0.015	**< 0.0001**
		**SNR (total)**		
Overall	4.449	0.060	4.332 to 4.566	**< 0.0001**
HD-YAS	8.541	0.124	8.298 to 8.785	**< 0.0001**
HD-CSF	5.450	0.182	5.094 to 5.806	**< 0.0001**
THD London	2.279	0.147	1.991 to 2.566	**< 0.0001**
THD Leiden	3.124	0.147	2.835 to 3.413	**< 0.0001**
THD Paris	2.311	0.149	2.019 to 2.604	**< 0.0001**
THD Vancouver	3.684	0.143	3.404 to 3.964	**< 0.0001**
		**SNR (GM)**		
Overall	1.650	0.047	1.558 to 1.741	**< 0.0001**
HD-YAS	4.223	0.097	4.033 to 4.414	**< 0.0001**
HD-CSF	3.531	0.142	3.252 to 3.809	**< 0.0001**
THD London	-0.606	0.115	-0.831 to -0.381	**< 0.0001**
THD Leiden	1.318	0.115	1.092 to 1.544	**< 0.0001**
THD Paris	-0.851	0.117	-1.080 to -0.623	**< 0.0001**
THD Vancouver	1.829	0.112	1.610 to 2.048	**< 0.0001**
		**SNR (WM)**		
Overall	9.751	0.140	9.476 to 10.026	**< 0.0001**
HD-YAS	19.376	0.292	18.802 to 19.949	**< 0.0001**
HD-CSF	11.067	0.428	10.229 to 11.905	**< 0.0001**
THD London	5.898	0.345	5.222 to 6.575	**< 0.0001**
THD Leiden	5.621	0.347	4.940 to 6.301	**< 0.0001**
THD Paris	6.010	0.351	5.322 to 6.699	**< 0.0001**
THD Vancouver	7.179	0.336	6.520 to 7.837	**< 0.0001**
		**SNR (CSF)**		
Overall	1.946	0.035	1.878 to 2.014	**< 0.0001**
HD-YAS	2.024	0.073	1.882 to 2.167	**< 0.0001**
HD-CSF	1.753	0.106	1.545 to 1.961	**< 0.0001**
THD London	1.544	0.086	1.376 to 1.712	**< 0.0001**
THD Leiden	2.433	0.086	2.264 to 2.601	**< 0.0001**
THD Paris	1.775	0.087	1.604 to 1.946	**< 0.0001**
THD Vancouver	2.044	0.083	1.881 to 2.207	**< 0.0001**

Data derived from linear mixed-effects models between unharmonized and harmonized metrics; adjusted MD represents the average marginal effect; bold values were statistically significant. CNR is defined between GM and WM. CJV reflect field inhomogeneity. The definition of noise in SNR is the within-tissue variance. CI, confidence interval; CJV, coefficient of joint variation; CNR, contrast-to-noise ratio; CSF, cerebrospinal fluid; GM, grey matter; HD, Huntington's disease; HD-CSF, HD Cerebrospinal Fluid Study; HD-YAS, HD Young Adult Study; MD, mean difference; SE, standard error; SNR, signal-to-noise ratio; THD, TRACK-HD study site; WM, white matter.

Supplementary Table 13: Variance explained by Principal Components in unharmonized and harmonized T1W MRI

**PCA Component**	**Variance explained by Principal Components (%)**	
	Unharmonized	Harmonized
**PC1**	97.61	35.68
**PC2**	0.59	15.78
**PC3**	0.41	10.02
**PC4**	0.32	8.95

PC, principal component; PCA, principal component analysis.

Supplementary Table 14: Variance explained by Principal Components in unharmonized and harmonized healthy control T1W MRI only

PCA Component	Variance explained by Principal Components (%)	
	Unharmonized	Harmonized
**PC1**	96.84	31.14
**PC2**	0.77	15.50
**PC3**	0.52	11.10
**PC4**	0.38	9.22

PC, principal component; PCA, principal component analysis.

Supplementary Table 15: QC outcomes for grey and white matter TPMs, both before and after harmonization

Study	Unharmonized			Harmonized		
	Scores 0-3 (n)	Score 4-5 (n)		Scores 0-3 (n)	Score 4-5 (n)	
		Non-systematic	Systematic		Non-systematic	Systematic
			**Grey matter**			
**HD-YAS**	3	3	117	120	3	0
**HD-CSF**	45	10	3	49	9	0
**TRACK-HD London**	85	4	0	87	2	0
**TRACK-HD Leiden**	81	7	0	81	7	0
**TRACK-HD Paris**	83	3	0	83	3	0
**TRACK-HD Vancouver**	87	7	0	85	9	0
**Total**	384	34	120	505	33	0
			**White matter**			
**HD-YAS**	6	0	117	123	0	0
**HD-CSF**	55	0	3	58	0	0
**TRACK-HD London**	87	2	0	89	0	0
**TRACK-HD Leiden**	88	0	0	88	0	0
**TRACK-HD Paris**	84	2	0	84	2	0
**TRACK-HD Vancouver**	94	0	0	94	0	0
**Total**	414	4	120	536	2	0

Segmentation QC ratings before and after harmonization by scanner, shown separately for grey matter and white matter TPMs before and after harmonization. Systematic errors refer to consistent, scanner-driven misclassification affecting scans from a given scanner, including grey matter over-segmentation and white matter under-segmentation; non-systematic errors refer to sporadic tissue misclassifications unrelated to scanner identity, most commonly meningeal over-segmentation. Grey and white matter, and unharmonized and harmonized counts are independent, as a participant may pass QC at one stage but not the other; the 501 participants taken forward for analysis represent those passing QC in both statuses across both tissue classes. Where either tissue class failed QC, all corresponding TPMs were excluded for that participant across both harmonization statuses. Scans scoring >3 due to systematic errors were retained in harmonization pipeline comparisons to characterize the full extent of segmentation failure and its resolution; all scans scoring >3 were excluded from biological preservation analyses (Section 3.4). HD, Huntington's disease; HD-CSF, HD Cerebrospinal Fluid Study; HD-YAS, HD Young Adult Study; QC, quality control; TPM, tissue probability map.

Supplementary Table 16: Scanner specific voxel-wise statistics between unharmonized and harmonized grey and white matter TPMs

Contrast (F or T contrast)	Total clusters	Largest cluster	Peak score	MNI coordinate	Atlas Location	p
	N	*n voxels*	*F/T-score*	*x, y, z (mm)*	*Grey matter: AAL3 White matter: HCPEx*	*FWE-corrected*
**Grey matter**						
**Main effect of harmonization (F)**	182	387	281.5	2, 26, -26	R rectus	< 0.0001
**Overall (T)**	88	518	13.5	-12, -30, -2	L Thalamus	< 0.0001
**HD-YAS (T)**	84	6491	25.93	8, -12, 15	R thalamus	< 0.0001
**HD-CSF (T)**	16	33	6.1	27, -58, -34	R cerebellum	< 0.0001
**THD London (T)**	29	2647	12.9	-14, -4, -32	L parahippocampal gyrus	< 0.0001
**THD Leiden (T)**	21	1214	13.5	-3, -8, -14	L substantia nigra	< 0.0001
**THD Paris (T)**	33	4334	11.32	8, -2, -15	R substantia nigra	< 0.0001
**THD Vancouver (T)**	12	759	10.36	8, 12, -22	R rectus	< 0.0001
**White matter**						
**Main effect of harmonization (F)**	68	3110	181	-14, -18, 18	L internal capsule	< 0.0001
**Overall (T)**	14	392	11.6	16, 20, -16	Undefined	< 0.0001
**HD-YAS (T)**	16	4582	21.09	14, 15, 54	Undefined	< 0.0001
**HD-CSF (T)**	32	56	10.35	6, -54, -18	Undefined	< 0.0001
**THD London (T)**	12	112	8.08	6, -28, -4	Undefined	< 0.0001
**THD Leiden (T)**	14	66	8.88	32, -2, 4	R internal capsule	< 0.0001
**THD Paris (T)**	15	29	7.4	8, -68, -28	Undefined	< 0.0001
**THD Vancouver (T)**	9	219	10.99	-30, -2, 8	L internal capsule	< 0.0001

Summary statistics were presented for significant clusters and peaks from grey matter and white matter scanner-wise voxel-based comparisons. Peak scores were from the largest significant cluster with the corresponding MNI coordinate. T contrasts were harmonized > unharmonized. Z-scores were omitted because all largest T- and F-values exceeded the finite range of the T/F→Z transformation, producing Z = Inf. Peak statistics were therefore reported using the original T- or F-values together with FWE-corrected p-values. AAL3, Automated Anatomical Labelling atlas 3; FWE, family-wise error; HCPEx, Extended Human Connectome Project multimodal parcellation atlas; HD, Huntington's disease; HD-CSF, HD Cerebrospinal Fluid Study; HD-YAS, HD Young Adult Study; L, left; MNI, Montreal Neurological Institute; R, right; THD, TRACK-HD study site.

Supplementary Table 17: Scanner specific volumetric differences between unharmonized and harmonized grey and white matter

	Tissue	Adjusted MD	SE	95% CI	p-value
**Overall volumetric differences**					
**Overall**	Grey matter	11.878	1.323	9.286 to 14.471	< 0.0001
	White matter	-17.623	0.837	-19.263 to -15.983	< 0.0001
**Scanner specific volumetric differences**					
**HD-YAS**	Grey matter	13.245	1.235	9.988 to 16.502	< 0.0001
	White matter	-15.554	0.781	-17.614 to -13.494	< 0.0001
**HD-CSF**	Grey matter	-38.778	1.823	-43.588 to -33.967	< 0.0001
	White matter	18.425	1.153	15.382 to 21.467	< 0.0001
**TRACK-HD London**	Grey matter	23.99	1.362	20.397 to 27.583	< 0.0001
	White matter	-17.513	0.861	-19.785 to -15.24	< 0.0001
**TRACK-HD Leiden**	Grey matter	12.429	1.395	8.748 to 16.11	< 0.0001
	White matter	-12.256	0.882	-14.584 to -9.928	< 0.0001
**TRACK-HD Paris**	Grey matter	24.799	1.387	21.139 to 28.459	< 0.0001
	White matter	-16.832	0.877	-19.146 to -14.518	< 0.0001
**TRACK-HD Vancouver**	Grey matter	7.895	1.364	4.296 to 11.495	< 0.0001
	White matter	-14.146	0.863	-16.422 to -11.87	< 0.0001

Values obtained from post-hoc pairwise comparisons of estimated marginal means (Bonferroni-corrected) to quantify overall and scanner-specific differences between unharmonized and harmonized raw grey and white matter volumes. CI, confidence interval; HD, Huntington's disease; HD-CSF, HD Cerebrospinal Fluid Study; HD-YAS, HD Young Adult Study; MD, mean difference; SE, standard error.

Supplementary Table 18: Variance and intraclass correlation of grey and white matter volume between unharmonized and harmonized data, by scanner

	Tissue	N	Mean (mL)		Standard deviation			ICC	
			Unharmonized	Harmonized	Unharmonized	Harmonized	Pitman-Morgan p	Har - unhar	95% CI
**HD-YAS**	Grey matter	120	704.8	717.0	58.2	60.0	0.1760	0.9700	0.9572 to 0.9790
	White matter	120	459.0	441.8	52.4	50.7	0.0450	0.9843	0.9776 to 0.9890
**HD-CSF**	Grey matter	48	653.5	615.2	73.3	76.9	0.1129	0.9776	0.9604 to 0.9874
	White matter	48	404.2	423.3	60.5	60.0	0.7467	0.9829	0.9697 to 0.9904
**TRACK-HD London**	Grey matter	85	657.6	681.6	64.8	64.6	0.8852	0.9833	0.9744 to 0.9891
	White matter	85	450.5	433.1	60.9	57.8	0.0001	0.9921	0.9879 to 0.9949
**TRACK-HD Leiden**	Grey matter	81	648.4	660.8	66.0	66.0	0.9438	0.9906	0.9855 to 0.9940
	White matter	81	435.5	423.2	57.8	55.3	0.0005	0.9929	0.9889 to 0.9954
**TRACK-HD Paris**	Grey matter	82	644.3	669.0	78.7	76.7	0.1392	0.9877	0.9810 to 0.9921
	White matter	82	442.6	425.6	62.8	58.4	< 0.0001	0.9908	0.9858 to 0.9941
**TRACK-HD Vancouver**	Grey matter	85	673.6	681.3	78.2	77.1	0.2816	0.9923	0.9883 to 0.9950
	White matter	85	451.8	437.4	54.0	50.0	< 0.0001	0.9904	0.9853 to 0.9938

N represents the number of subjects with both unharmonized and harmonized measurements available (complete pairs) for each scanner and tissue class. The Pitman-Morgan test assessed equality of variance between unharmonized and harmonized measurements via the correlation between the pairwise mean [(unharmonized + harmonized)/2] and the pairwise difference (harmonized − unharmonized); rejection of the null indicates a significant change in variance. ICC was estimated using a two-way mixed-effects, consistency-type model. Bold values indicate p < 0.05. CI, confidence interval; HD, Huntington's disease; ICC, intraclass correlation coefficient; SD, standard deviation.

Supplementary Table 19: Within disease-specific voxel-wise statistics between unharmonized and harmonized grey and white matter TPMs

Contrast (F or T contrast)	Total clusters	Largest cluster	Peak score	MNI coordinate	Atlas Location	p
	N	*n voxels*	*F/T-score*	*x, y, z (mm)*	*Grey matter: AAL3 White matter: HCPEx*	*FWE-corrected*
			**Grey matter**			
**Main effect of harmonization (F)**	14	162	88.89	-32, -4, 3	L putamen	< 0.0001
**Overall (T)**	14	589	7.27	6, 2, -10	R nuclear accumbens	< 0.0001
**Healthy control (T)**	26	3053	11.65	6, 2, -10	R nuclear accumbens	< 0.0001
**pre-CMD (T)**	28	1992	10.51	8, 2, -10	R nuclear accumbens	< 0.0001
**post-CMD (T)**	24	743	9.19	8, 2, -10	R nuclear accumbens	< 0.0001
			**White matter**			
**Main effect of harmonization (F)**	37	290	108.4	21, 16, 2	R internal capsule	< 0.0001
**Overall (T)**	5	120	9.33	-32, -3, 8	L internal capsule	< 0.0001
**Healthy control (T)**	13	201	13.2	-32, -3, 8	L internal capsule	< 0.0001
**pre-CMD (T)**	13	132	11.08	-32, -3, 8	L internal capsule	< 0.0001
**post-CMD (T)**	23	42	9.05	4, -54, -18	Undefined	< 0.0001

Summary statistics were presented for significant clusters and peaks from grey matter and white matter within-disease voxel-based comparisons with those with > 3 on the QC severity rating score excluded (N = 379). Peak scores are from the largest significant cluster with the corresponding MNI coordinate. T contrasts are harmonized > unharmonized. Z-scores were omitted because all largest T- and F-values exceeded the finite range of the T/F→Z transformation, producing Z = Inf. Peak statistics are therefore reported using the original T- or F-values together with FWE-corrected p-values. AAL3, Automated Anatomical Labelling atlas 3; FWE, family-wise error; HCPEx, Extended Human Connectome Project multimodal parcellation atlas; L, left; MNI, Montreal Neurological Institute; post-CMD, pwHD after clinical motor diagnosis; pre-CMD, pwHD before clinical motor diagnosis; QC, quality control; R, right.

Supplementary Table 20: Within disease specific volumetric differences between unharmonized and harmonized grey and white matter

	Tissue	Adjusted MD	SE	95% CI	p-value
		**Within disease specific volumetric differences**			
**Healthy Controls**	Grey matter	4.212	1.572	0.448 to 7.975	0.0221
	White matter	-12.569	1.049	-15.081 to -10.057	< 0.0001
**pre-CMD**	Grey matter	6.061	1.574	2.294 to 9.829	0.0004
	White matter	-10.842	1.05	-13.357 to -8.327	< 0.0001
**post-CMD**	Grey matter	8.424	1.61	4.57 to 12.278	< 0.0001
	White matter	-6.75	1.075	-9.323 to -4.177	< 0.0001

Values obtained from post-hoc pairwise comparisons of estimated marginal means (Bonferroni-corrected) with those with > 3 on the QC severity rating score excluded (N = 379) to quantify disease-specific differences between unharmonized and harmonized grey and white matter volumes. CI, confidence interval; HD, Huntington’s disease; MD, mean difference; post-CMD, pwHD after clinical motor diagnosis; pre-CMD, pwHD before clinical motor diagnosis; QC, quality control; SE, standard error.

Supplementary Table 21: Variance and intraclass correlation of grey and white matter volume between unharmonized and harmonized data, by disease group

	Tissue	N	Mean		Standard deviation			ICC	
			Unharmonized	Harmonized	Unharmonized	Harmonized	Pitman-Morgan p	Har - unhar	95% CI
**Control**	Grey matter	127	679.2	689.6	66.3	67.6	0.4182	0.9641	0.9493 to 0.9746
	White matter	127	461.7	446.7	58.7	54.8	0.0010	0.9716	0.9599 to 0.9799
**PreHD**	Grey matter	125	675.7	687.7	67.5	67.8	0.8915	0.9617	0.9459 to 0.9730
	White matter	125	448.0	435.0	60.8	57.1	0.0004	0.9802	0.9719 to 0.9861
**HD**	Grey matter	127	611.2	620.6	63.0	67.8	0.0547	0.9043	0.8668 to 0.9317
	White matter	127	411.4	405.7	52.2	49.0	0.0097	0.9593	0.9427 to 0.9712

N represents the number of subjects with both unharmonized and harmonized measurements available (complete pairs) for each scanner and tissue class with those with > 3 on the QC severity rating score excluded (N = 379). The Pitman-Morgan test assessed equality of variance between unharmonized and harmonized measurements via the correlation between the pairwise mean [(unharmonized + harmonized)/2] and the pairwise difference (harmonized − unharmonized); rejection of the null indicates a significant change in variance. ICC was estimated using a two-way mixed-effects, consistency-type model. Bold values indicate p < 0.05. CI, confidence interval; HD, Huntington's disease; ICC, intraclass correlation coefficient; SD, standard deviation.

Supplementary Table 22: Voxel-wise between disease group differences in unharmonized and harmonized T1W MRI

Contrast	Condition	Total clusters	Largest cluster	Peak score	MNI coordinate	Atlas Location	p
		N	*n voxels*	*T-score*	*x, y, z (mm)*	*Grey matter: AAL3 White matter: HCPEx*	*FWE-corrected*
				**Grey matter**			
**HC > pre-CMD**	Unharmonized	18	1489	11.67	30, -10, -8	R putamen	< 0.0001
	Harmonized	12	3862	14.51	32, -10, -9	R putamen	< 0.0001
**HC > post-CMD**	Unharmonized	167	48505	24.37	22, 10, 2	R putamen	< 0.0001
	Harmonized	165	39360	25.3	27, 9, 4	R putamen	< 0.0001
				**White matter**			
**HC > pre-CMD**	Unharmonized	39	3706	9.03	-24, -6, -4	L internal capsule	< 0.0001
	Harmonized	32	3080	9.21	-24, -6, -4	L internal capsule	< 0.0001
**HC > post-CMD**	Unharmonized	9	102682	20.01	21, 3, -2	R internal capsule	< 0.0001
	Harmonized	6	90751	20.48	-21, 0, -2	L internal capsule	< 0.0001

Summary statistics were presented for significant clusters and peaks from grey matter and white matter disease group voxel-based comparisons with those with > 3 on QC severity scale excluded (N = 379). Peak scores were from the largest significant cluster with the corresponding MNI coordinate. T-contrasts compare healthy controls to pre-CMD and post-CMD participants. Z-scores were omitted because all largest T- and F-values exceeded the finite range of the T/F→Z transformation, producing Z = Inf. Peak statistics were therefore reported using the original T- or F-values together with FWE-corrected p-values. AAL3, Automated Anatomical Labelling atlas 3; FWE, family-wise error; HC, healthy controls; HCPEx, Extended Human Connectome Project multimodal parcellation atlas; L, left; MNI, Montreal Neurological Institute; post-CMD, pwHD after clinical motor diagnosis; pre-CMD, pwHD before clinical motor diagnosis; R, right.

Supplementary Table 23: Volumetric post-hoc disease group differences in grey and white matter before and after harmonization

Comparison	Condition	Adj. MD	SE	95% CI	p
		**Grey matter**			
**HC versus pre-CMD**	Unharmonised	-0.22	0.36	-1.11 to 0.66	1.000
	Harmonised	-0.1	0.36	-0.98 to 0.79	1.000
**HC versus post-CMD**	Unharmonised	-3.93	0.36	-4.82 to -3.04	< 0.0001
	Harmonised	-3.61	0.36	-4.5 to -2.72	< 0.0001
		**White matter**			
**HC versus pre-CMD**	Unharmonised	-0.92	0.23	-1.49 to -0.35	0.0002
	Harmonised	-0.81	0.23	-1.38 to -0.24	0.0016
**HC versus post-CMD**	Unharmonised	-2.64	0.23	-3.22 to -2.84	< 0.0001
	Harmonised	-2.27	0.23	-2.84 to -1.69	< 0.0001

Values obtained from post-hoc pairwise comparisons of estimated marginal means (Bonferroni-corrected) to quantify disease-group difference in grey and white volumes before and after harmonisation with those with > 3 on the QC severity rating score excluded (N = 379). Volumes are % of TIV (volume/TIV * 100). CI, confidence interval; HC, healthy control; MD, mean difference; post-CMD, pwHD after clinical motor diagnosis; pre-CMD, pwHD before clinical motor diagnosis; QC, quality control; SE, standard error.

4 Supplementary Figures

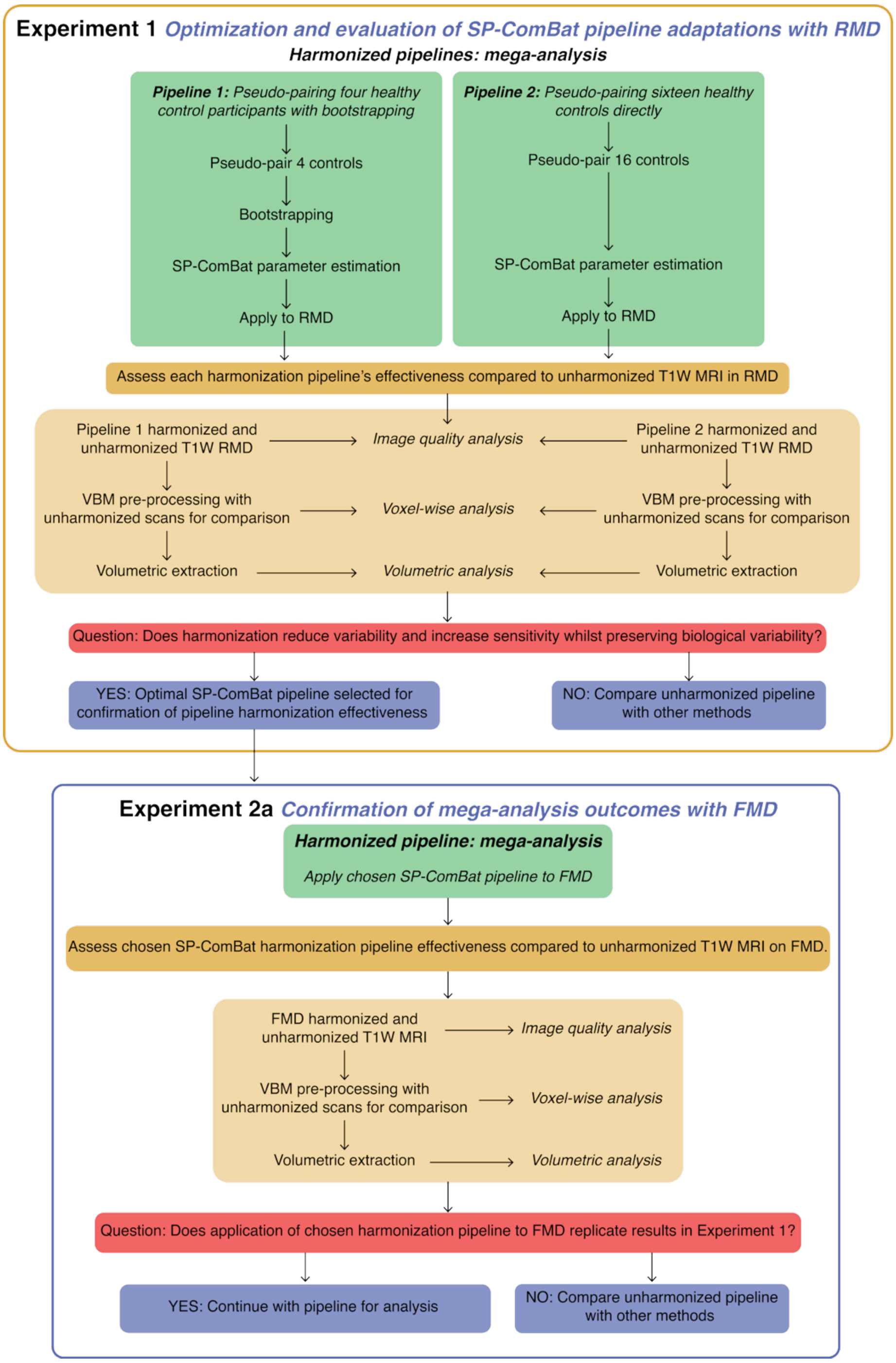

Supplementary Figure 1: Pre-registered study design for unpaired SP-ComBat harmonization pipelines. The study was pre-registered prior to analysis and reports results from Experiments 1 and 2a. Experiment 1 involved developing and optimizing two SP-ComBat pipelines adapted for cross-sectional unpaired data and evaluating their ability to harmonize multi-study T1W MRI in the absence of traveling subjects. Experiment 2a applied the pipeline selected in Experiment 1 to the full multi-study dataset (FMD) to replicate and further assess harmonization effectiveness. The go/no-go decision point is indicated by the red box. FMD, full multi-study dataset; MRI, magnetic resonance imaging; RMD, representative multi-study dataset; SP-ComBat, Superpixel-ComBat; T1W, T1-weighted; VBM, voxel-based morphometry

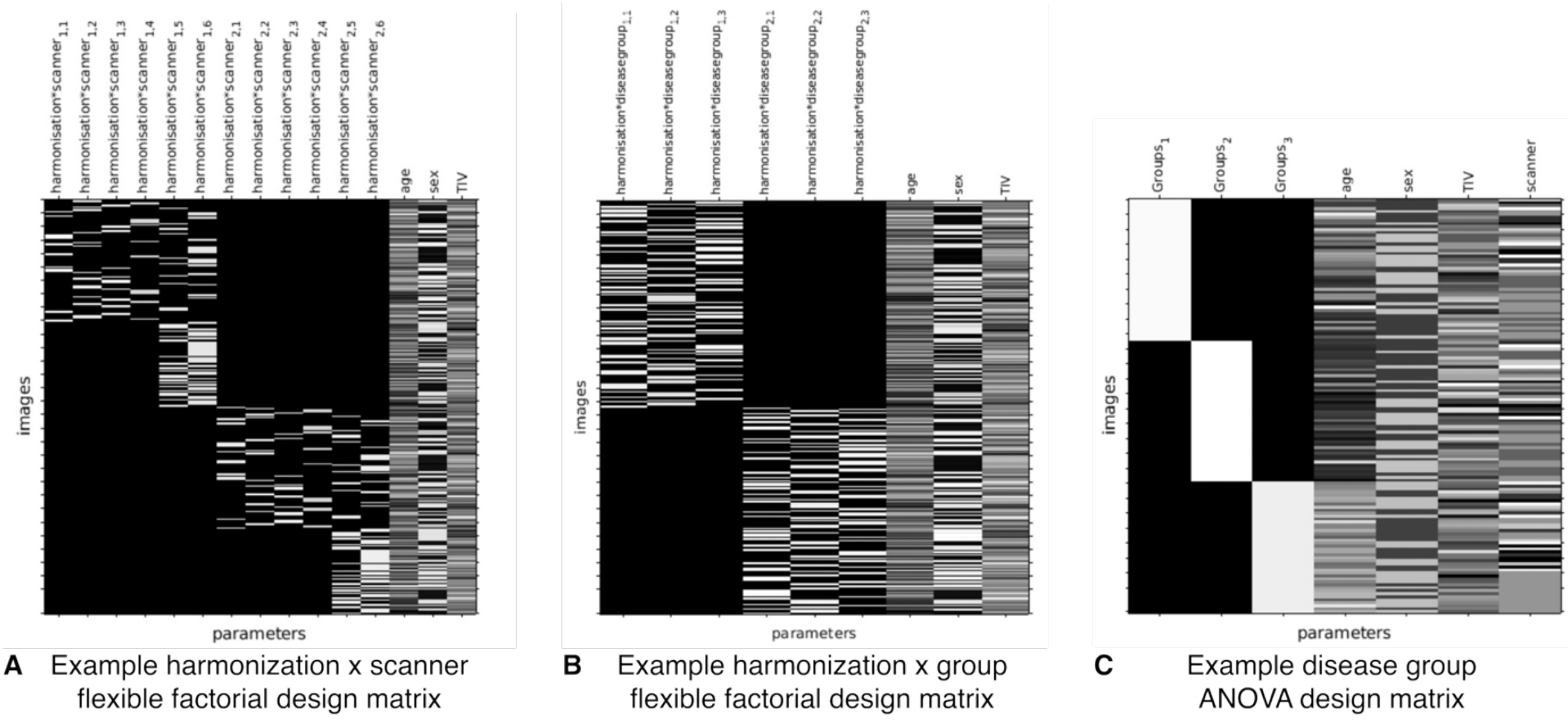

Supplementary Figure 2: SPM12 general linear model design matrices for voxel-wise harmonization and disease group analyses. Flexible factorial GLM design matrices for comparing unharmonized and harmonized grey and white matter TPMs across scanner type (A) and disease group (B), and an example one-way ANOVA design matrix for disease group differences (C). In (A), 'harmonization' denotes unharmonized (1) or harmonized (2) status and 'scanner' denotes TRACK-HD London (1), TRACK-HD Leiden (2), TRACK-HD Paris (3), TRACK-HD Vancouver (4), HD-YAS (5), or HD-CSF (6). In (B), 'harmonization' denotes unharmonized (1) or harmonized (2) status and 'disease group' denotes healthy controls (1), pre-CMD (2), and post-CMD (3). In (C), disease groups are healthy controls (1), pre-CMD (2), and post-CMD (3); scanner was included as a covariate in unharmonized analyses only. HD, Huntington's disease; HD-CSF, HD Cerebrospinal Fluid Study; HD-YAS, HD Young Adult Study; GLM, general linear model; HD, Huntington's disease; post-CMD, pwHD after clinical motor diagnosis; pre-CMD, pwHD before clinical motor diagnosis; TPM, tissue probability map.

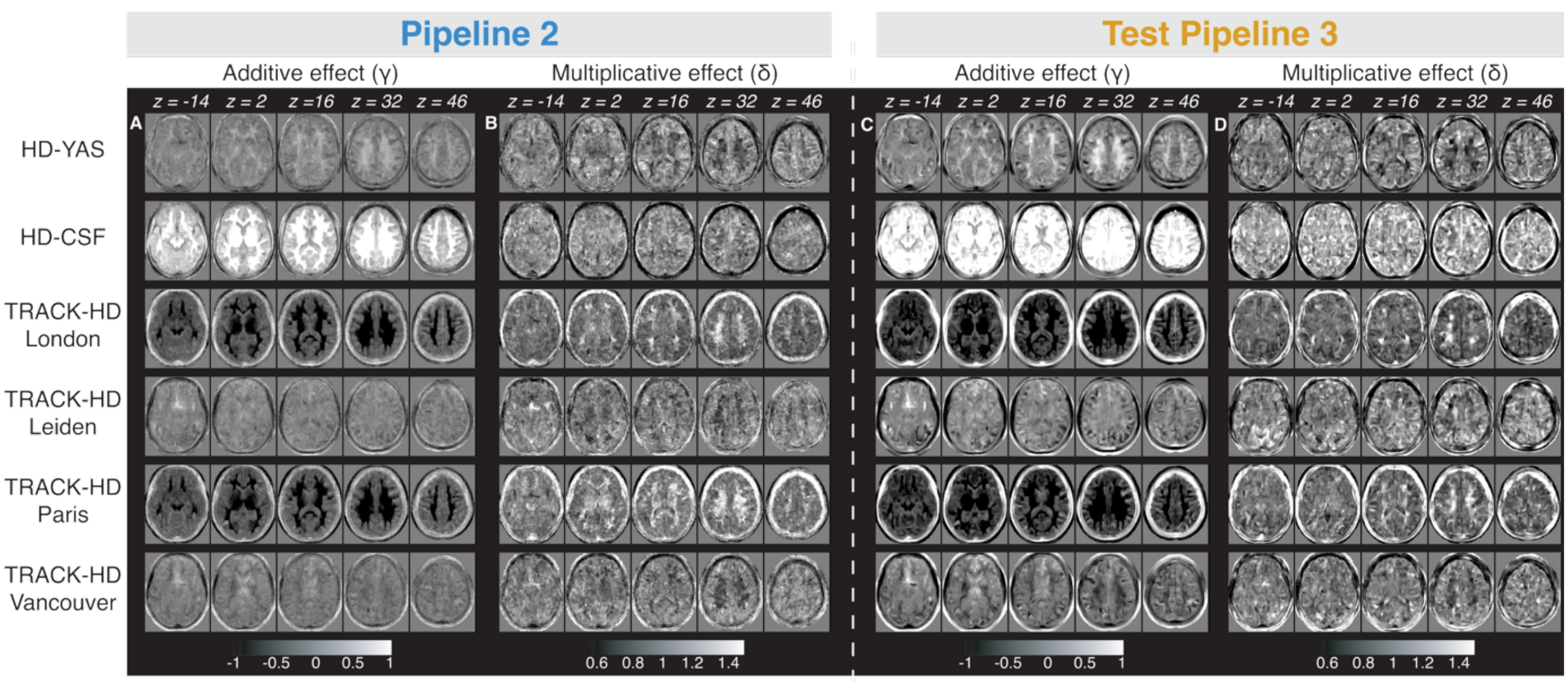

Supplementary Figure 3: Scanner-specific additive and multiplicative parametric maps for Pipeline 2 and a bootstrapped variant (Pipeline 3). Mean parametric maps showing additive (γ) and multiplicative (δ) effects estimated from Pipeline 2's 16 directly matched pseudo-pairs (A–B) and from Pipeline 3, in which the same 16 pseudo-pairs were bootstrapped to 25 resampled pairs (C–D), matching the resampling procedure applied to Pipeline 1. Maps were displayed in MRIcron with fixed intensity scales (γ: −1 to 1; δ: 0.5 to 1.5). z, axial slice position in MNI space. HD, Huntington's disease; HD-CSF, HD Cerebrospinal Fluid Study; HD-YAS, HD Young Adult Study.

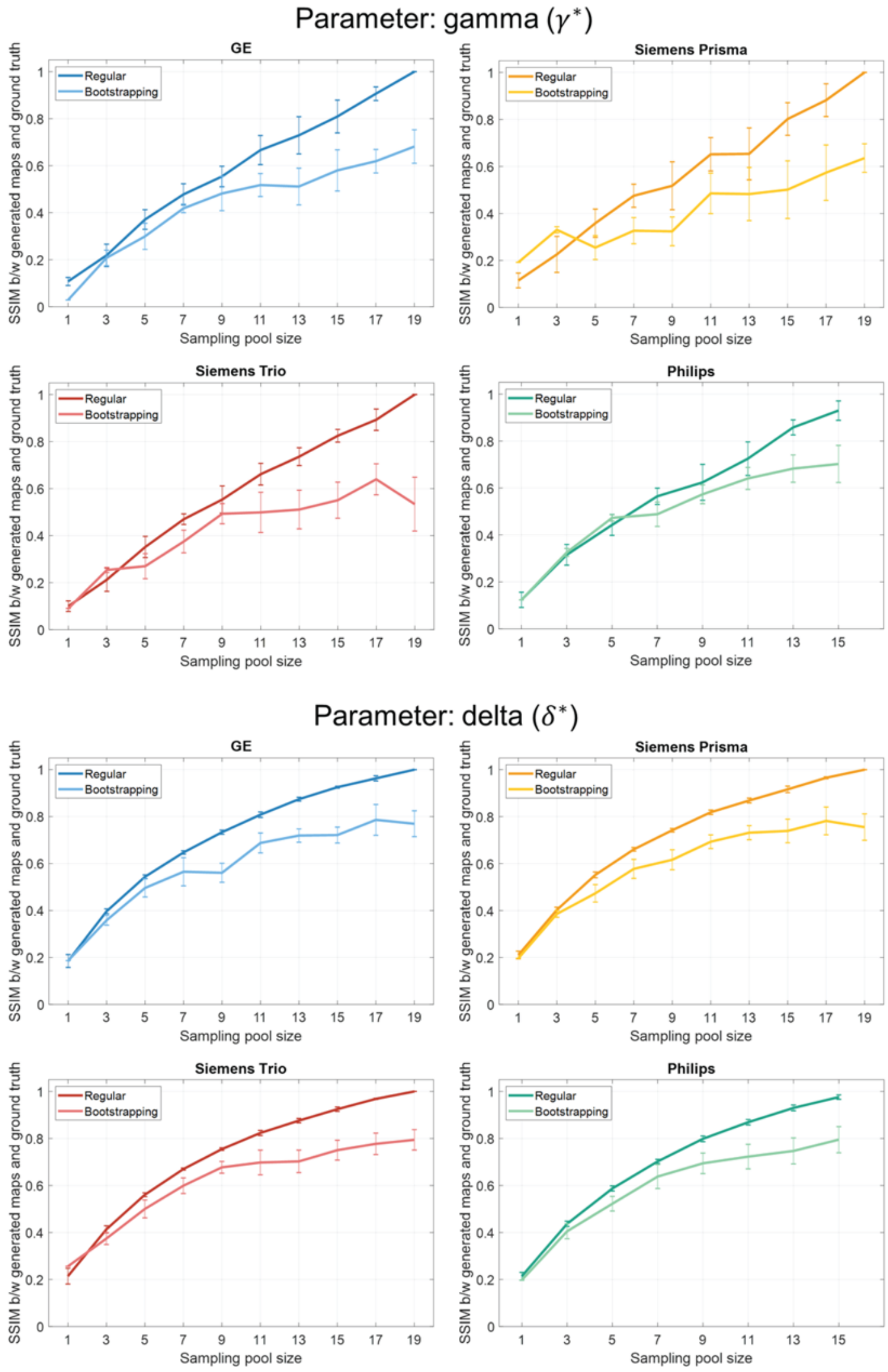

Supplementary Figure 4: Convergence of SP-ComBat parametric maps with increasing subject-pool size under regular permutation versus bootstrap resampling, across four independent scanner platforms. Structural similarity index measure (SSIM) between aggregated parametric maps and ground-truth reference maps, estimated from the full available sample for each scanner, plotted against subject-pool size for additive (gamma; γ) and multiplicative (delta; δ) effects: GE, Siemens Prisma, Siemens Trio, and Philips. At each pool size, aggregated maps were generated by either direct averaging of traveling subjects ("regular permutation") or bootstrap resampling with replacement to a fixed synthetic cohort size of 19 ("bootstrapping") and compared against the ground truth using SSIM. Points and error bars represent mean ± SD across 8 repetitions per pool size. Data are from an independent dataset underlying the original SP-ComBat validation study (Chen et al. 2024), distinct from the HD-YAS, HD-CSF, and TRACK-HD scanners used in the present study. SD, standard deviation; SP-ComBat, Superpixel-ComBat; SSIM, structural similarity index measure.

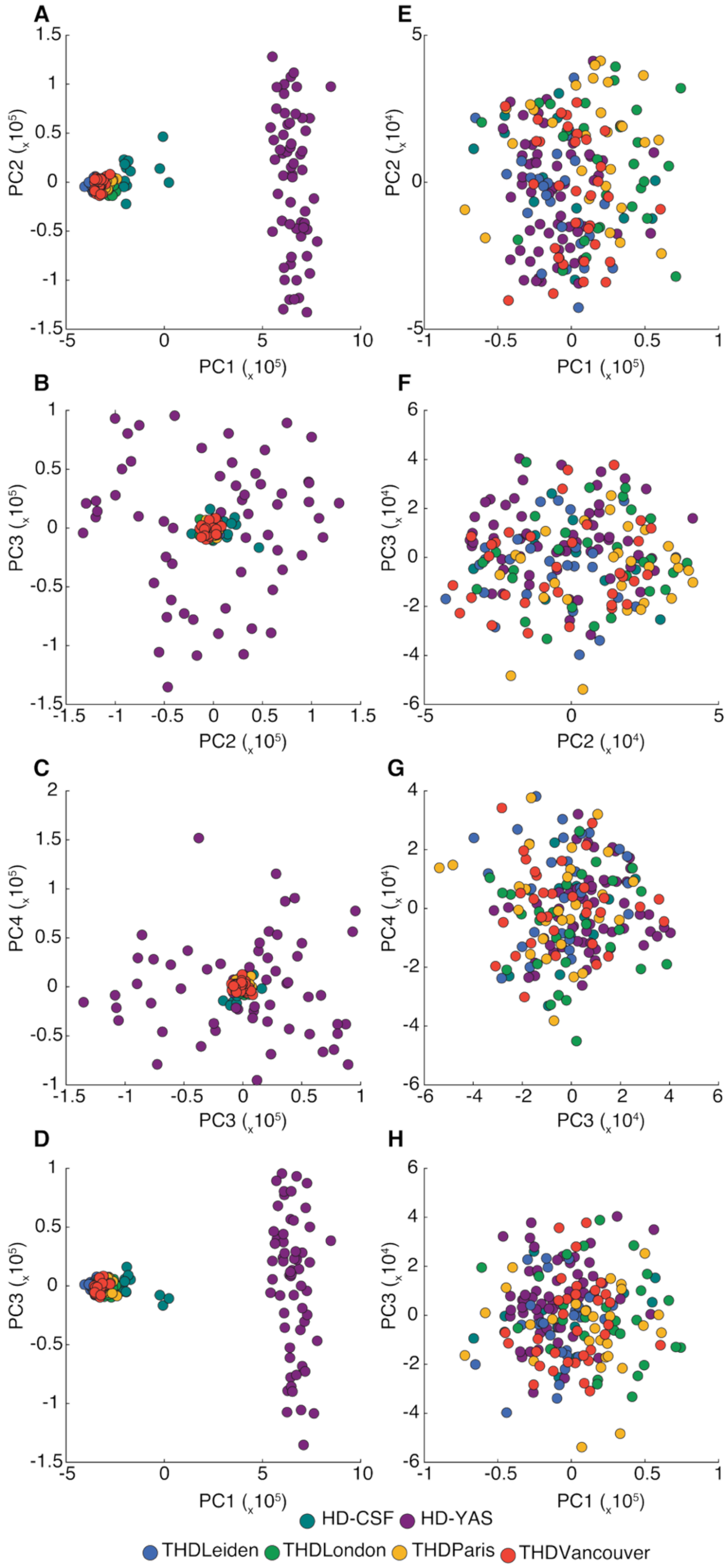

Supplementary Figure 5: Principal component analysis of intracranially masked voxel intensities in healthy controls before and after SP-ComBat harmonization. PCA applied to intracranially masked voxels from healthy control T1W MRI scans before harmonization (A–D) and after SP-ComBat harmonization (E–H), confirming that scanner-driven clustering observed in the full dataset reflected scanner differences rather than disease-related variance. HD, Huntington's disease; HD-CSF, HD Cerebrospinal Fluid Study; HD-YAS, HD Young Adult Study; PC, principal component; PCA, principal component analysis; THD, TRACK-HD study site.

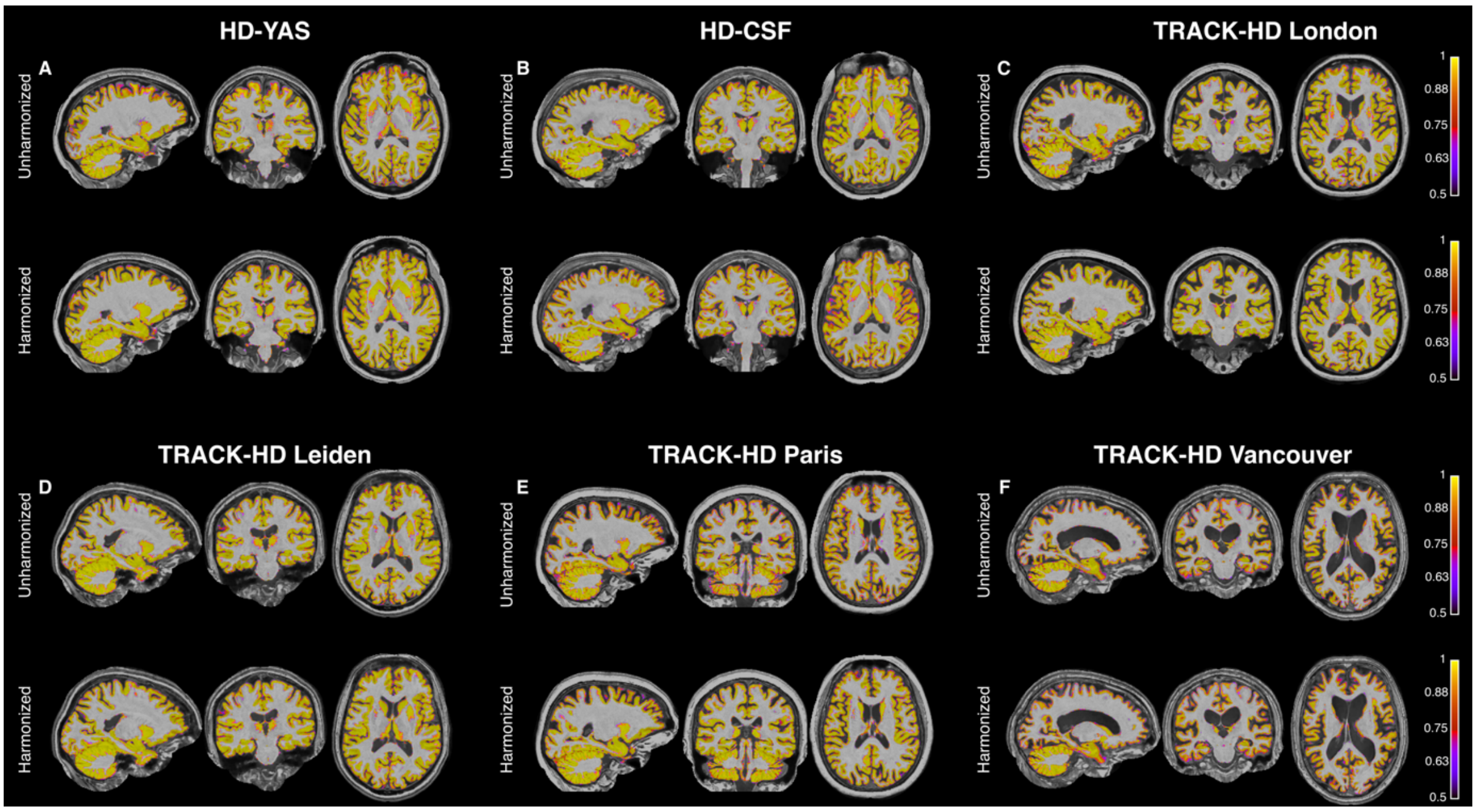

Supplementary Figure 6: Scanner-specific grey matter segmentations before and after SP-ComBat harmonization across all six scanners. Grey matter segmentations overlaid on native-space T1W MRI for representative scans from HD-YAS (A), HD-CSF (B), TRACK-HD London (C), TRACK-HD Leiden (D), TRACK-HD Paris (E), and TRACK-HD Vancouver (F), shown before and after harmonization. Images were displayed in three-plane orthogonal view with representative sagittal, coronal, and axial slices. Both unharmonized and harmonized segmentations were overlaid on the harmonized T1W MRI to enable direct visual comparison on a common reference image. HD, Huntington's disease; HD-CSF, HD Cerebrospinal Fluid Study; HD-YAS, HD Young Adult Study.

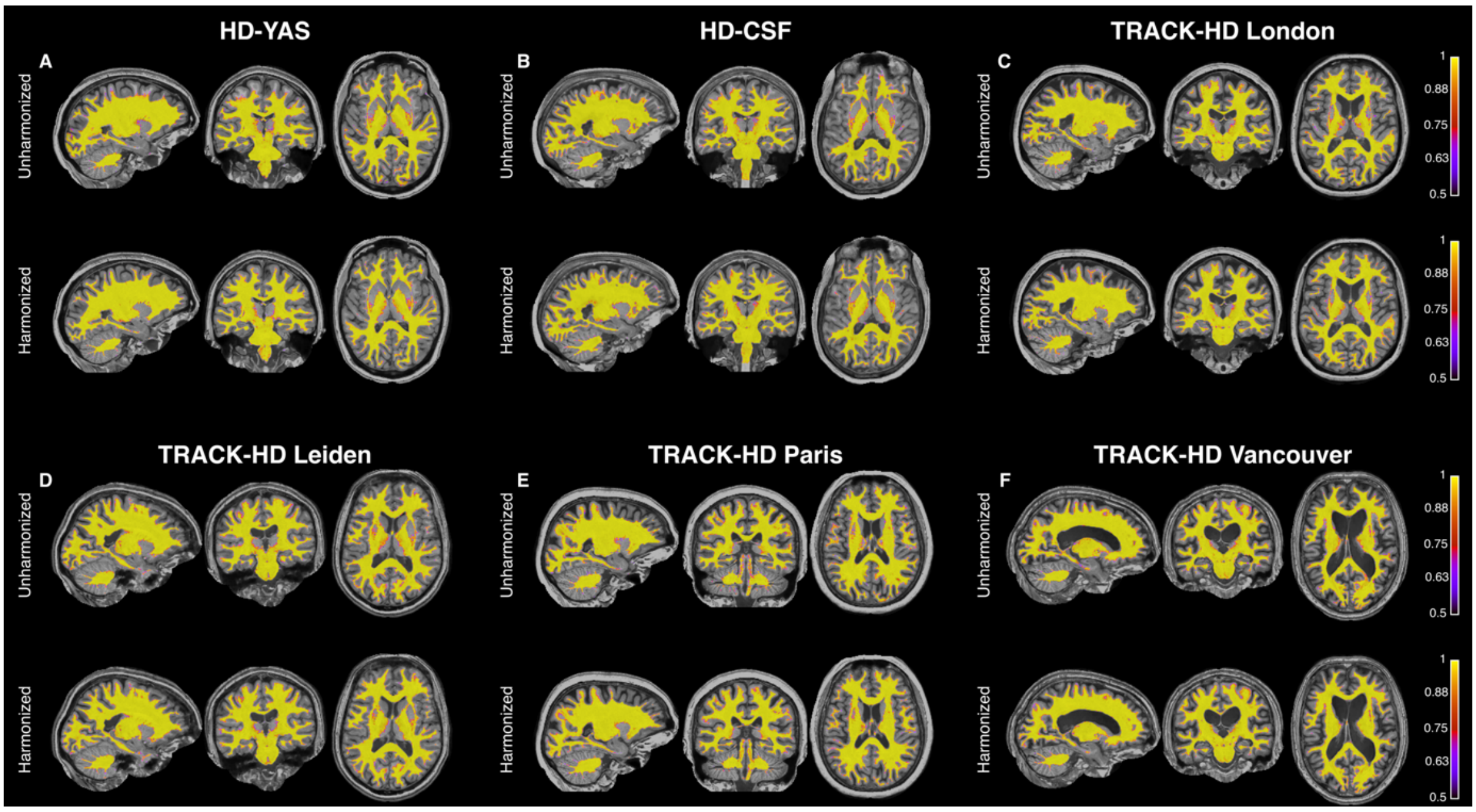

Supplementary Figure 7: Scanner-specific white matter segmentations before and after SP-ComBat harmonization across all six scanners. White matter segmentations overlaid on native-space T1W MRI for representative scans from HD-YAS (A), HD-CSF (B), TRACK-HD London (C), TRACK-HD Leiden (D), TRACK-HD Paris (E), and TRACK-HD Vancouver (F), shown before and after harmonization. Images were displayed in three-plane orthogonal view with representative sagittal, coronal, and axial slices. Both unharmonized and harmonized segmentations were overlaid on the harmonized T1W MRI to enable direct visual comparison on a common reference image. HD, Huntington's disease; HD-CSF, HD Cerebrospinal Fluid Study; HD-YAS, HD Young Adult Study.

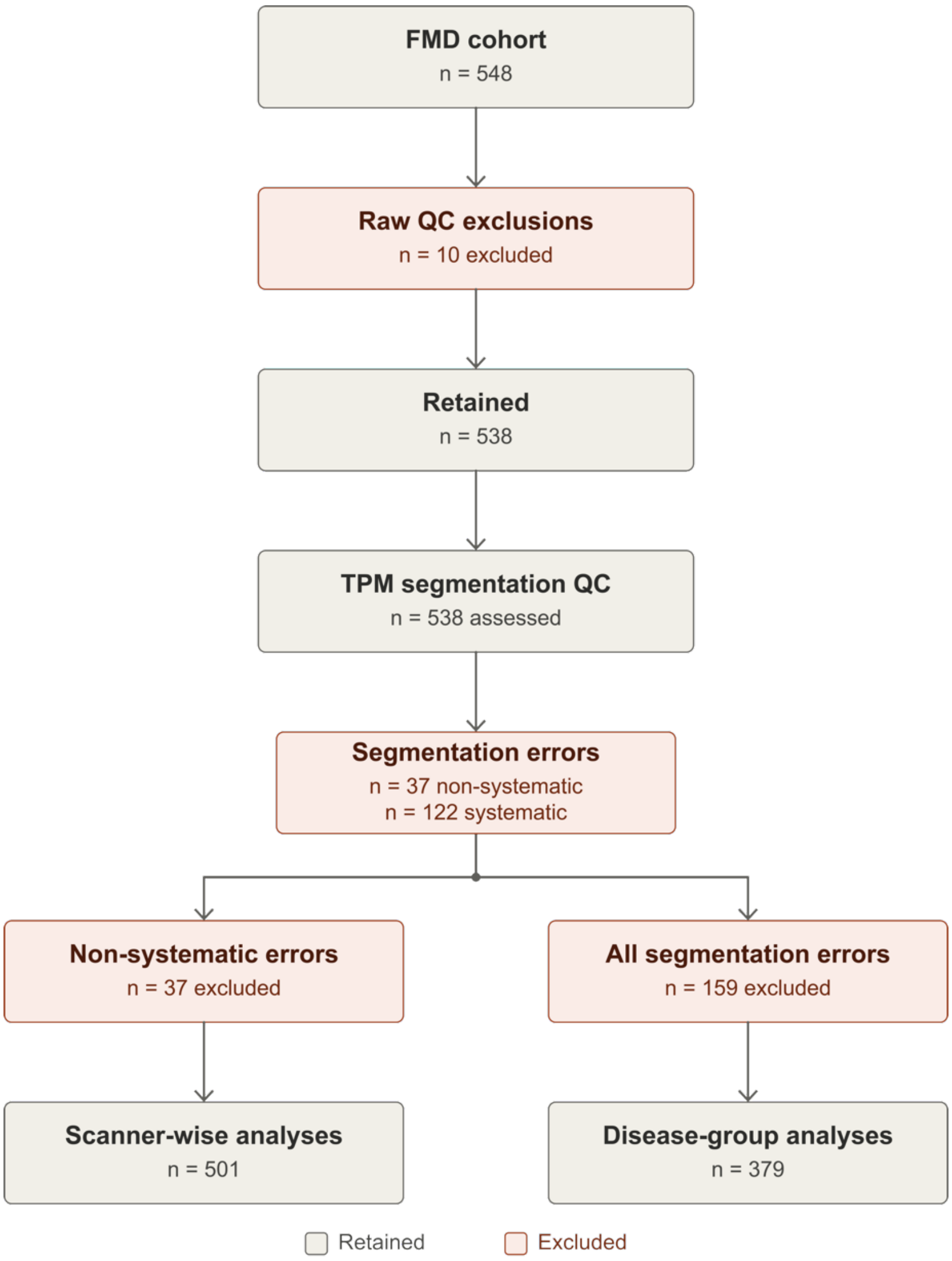

Supplementary Figure 8: Sample size flow through quality-control and analysis stages for the full multi-study dataset (FMD). Of 548 participants with available T1W MRI, 10 were excluded following raw-scan visual QC (severity rating >3, or evidence of non-HD pathology; Supplementary Table 2), leaving 538 scans for TPM segmentation QC (Supplementary Table 6). For scanner-specific voxel-wise and volumetric analyses, only TPMs failing QC due to non-systematic errors were excluded (n = 37), yielding 501 matched unharmonized/harmonized TPMs; TPMs with systematic scanner-driven errors were retained at this stage to characterize the extent of segmentation failure and its resolution by harmonization. For disease-group-specific analyses, TPMs failing QC due to either systematic or non-systematic errors were excluded (159 total), yielding the final analysis sample of 379 participants (Supplementary Table 15). FMD, full multi-study dataset; QC, quality control; T1W, T1-weighted; TPM, tissue probability map.

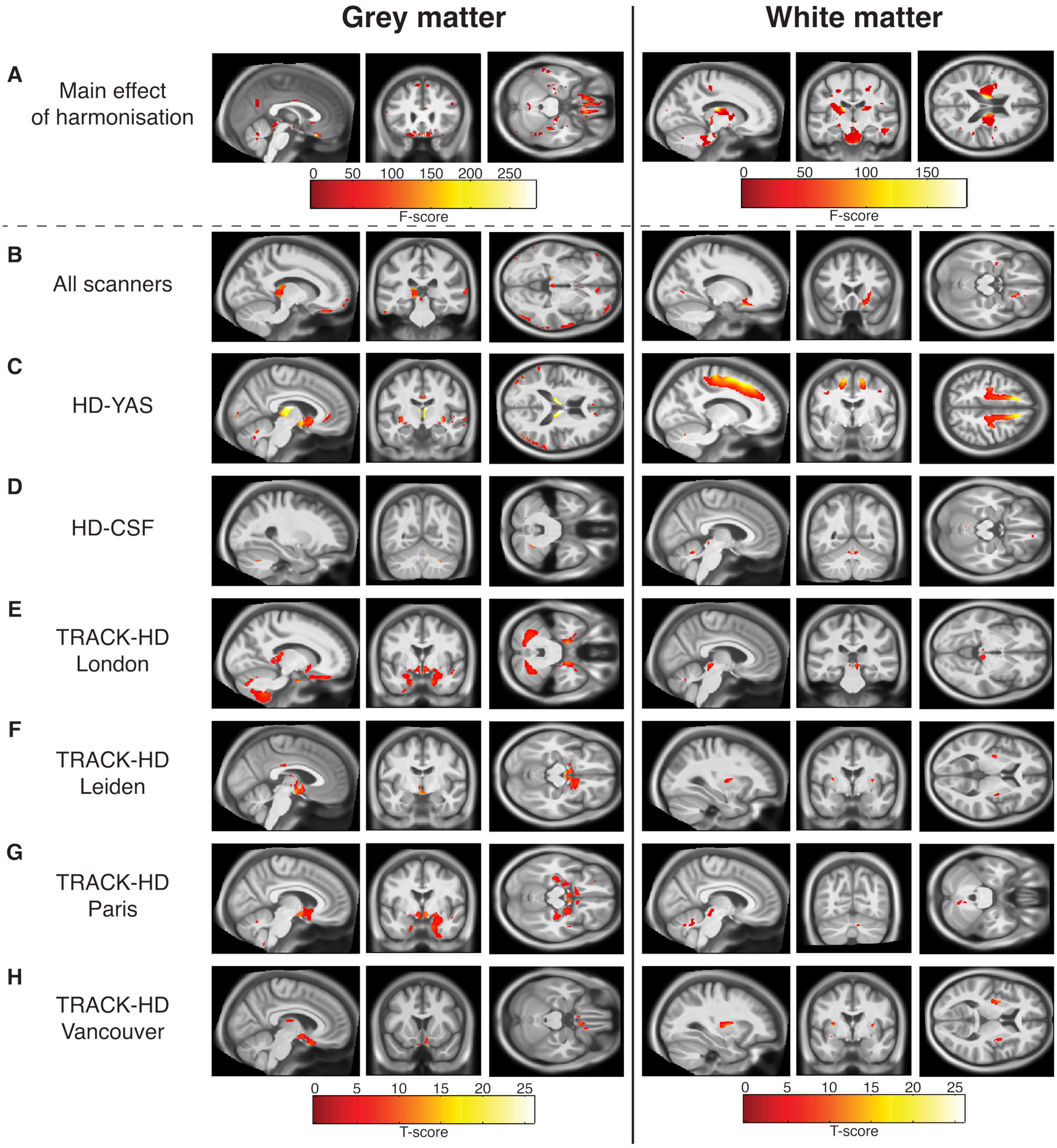

Supplementary Figure 9: Voxel-wise statistical parametric maps of grey and white matter differences between harmonized and unharmonized TPMs across all scanners. Statistical parametric maps showing grey and white matter differences between harmonized and unharmonized TPMs for the overall F-contrast (A), the overall T-contrast for harmonized > unharmonized (B), and scanner-specific T-contrasts for HD-YAS (C), HD-CSF (D), TRACK-HD London (E), TRACK-HD Leiden (F), TRACK-HD Paris (G), and TRACK-HD Vancouver (H). Significant clusters were overlaid onto sagittal, axial, and coronal sections of the study population-average brain; slices were selected at the contrast global maximum. The main effect of harmonization was displayed as F-scores; scanner-specific contrasts were displayed as T-scores. All comparisons were displayed on the same T-score scale (0–25); threshold $p < 0.05$, voxel-wise FWE-corrected. FWE, family-wise error; HD, Huntington's disease; HD-CSF, HD Cerebrospinal Fluid Study; HD-YAS, HD Young Adult Study; TPMs, tissue probability maps.

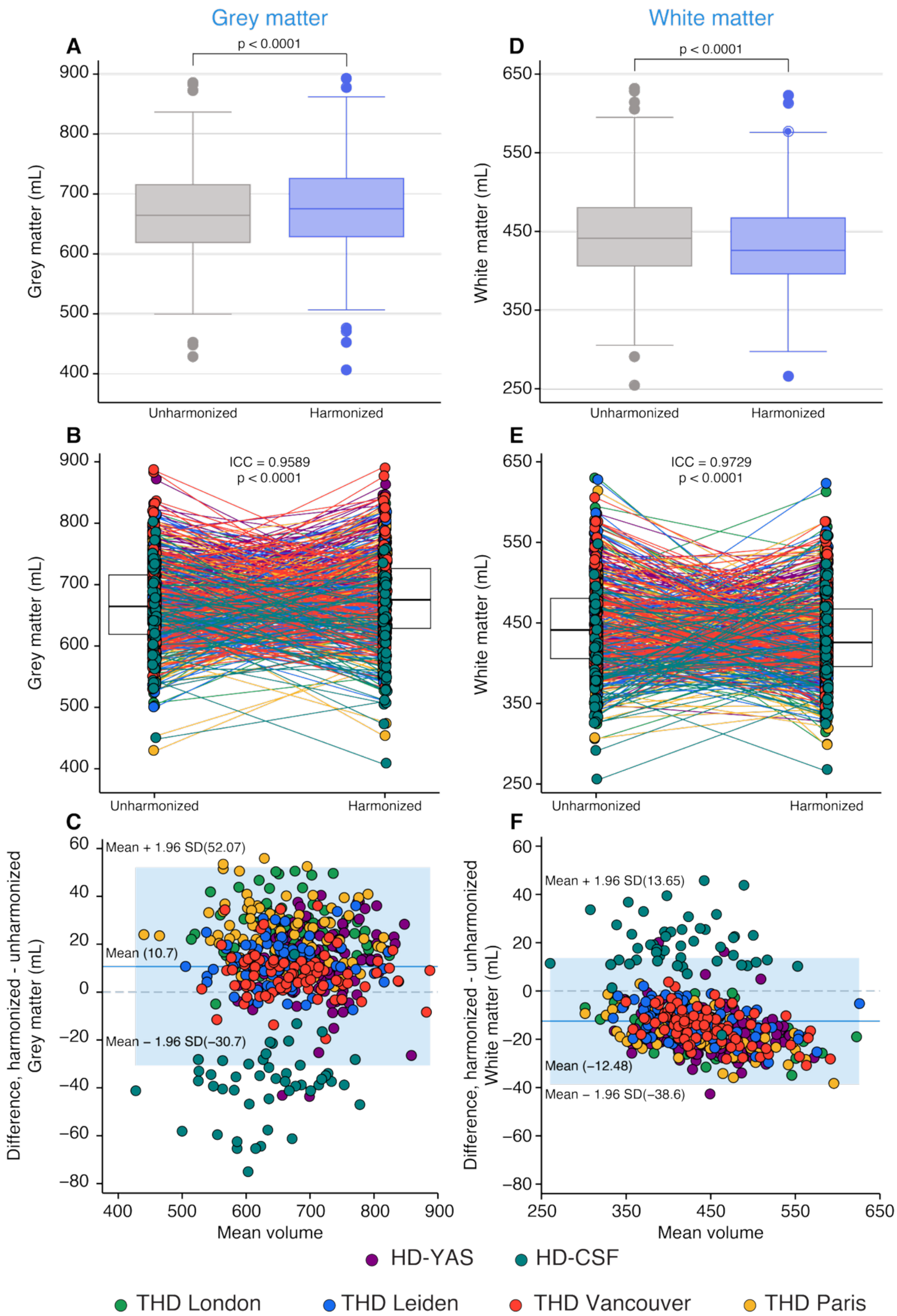

Supplementary Figure 10: Agreement between unharmonized and harmonized absolute grey and white matter volumes across all scanners. A and D) Differences between unharmonized and harmonized volumes in A) grey and D) whiter matter. P-values obtained from mixed-effects models including harmonization status and its interactions with scanner and disease group as fixed effects, with subject modelled as a random intercept. Boxes show first and third quartiles, the central band shows the median, and the whiskers show data within 1.5 IQR of the median. B and E) Spaghetti plots overlaid on box plots representing the agreement between volumetric measurements before and after harmonization in B) grey, and E) white matter. C and F) Bland-Altman plots for agreement between unharmonized and harmonized volumes in C) grey and F) white matter with the 95% limits of agreement represented by the limits of the light blue shaded region; dark blue lines represent the mean difference; dashed line represents y=0. HD, Huntington's disease; HD-CSF, HD Cerebrospinal Fluid Study; HD-YAS, HD Young Adult Study; ICC, intra-class correlation; THD, TRACK-HD study site; TPM, tissue probability map.

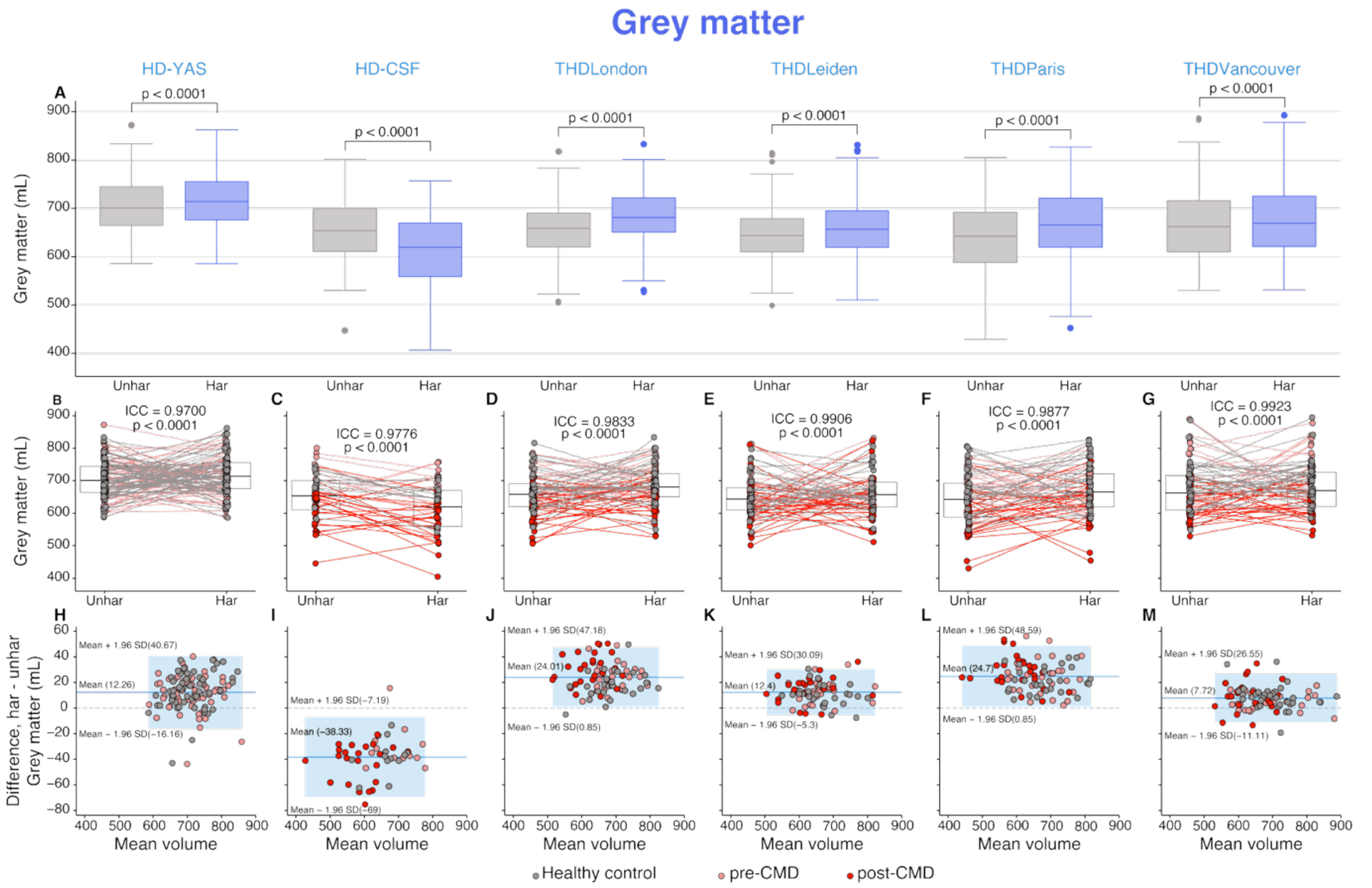

Supplementary Figure 11: Scanner-specific agreement between unharmonized and harmonized grey matter volumes. A) Differences between unharmonized and harmonized grey matter volumes for each scanner. P-values extracted from Bonferroni-corrected post-hoc pairwise comparisons of estimated marginal means derived from the mixed-effects model (fixed effects: harmonization × scanner × disease group; random intercept: subject). Boxes show first and third quartiles, the central band shows the median, and the whiskers show data within 1.5 IQR of the median. B-G) Spaghetti plots overlaid on box plots representing the agreement between volumetric measurements before and after harmonization in grey matter for B) HD-YAS, C) HD-CSF, D) TRACK-HD London, E) TRACK-HD Leiden, F) TRACK-HD Paris, and G) TRACK-HD Vancouver. H-M) Bland-Altman plots for agreement between unharmonized and harmonized volumes in grey matter for H) HD-YAS, I) HD-CSF, J) TRACK-HD London, K) TRACK-HD Leiden, L) TRACK-HD Paris, and M) TRACK-HD Vancouver with the 95% limits of agreement represented by the limits of the light blue shaded region; dark blue lines represent the mean difference; dashed line represents y=0. Har, harmonized; HD, Huntington's disease; HD-CSF, HD Cerebrospinal Fluid Study; HD-YAS, HD Young Adult Study; ICC, intra-class correlation; post-CMD, pwHD after clinical motor diagnosis; pre-CMD, pwHD before clinical motor diagnosis; THD, TRACK-HD study site; TPM, tissue probability map; unhar, unharmonized.

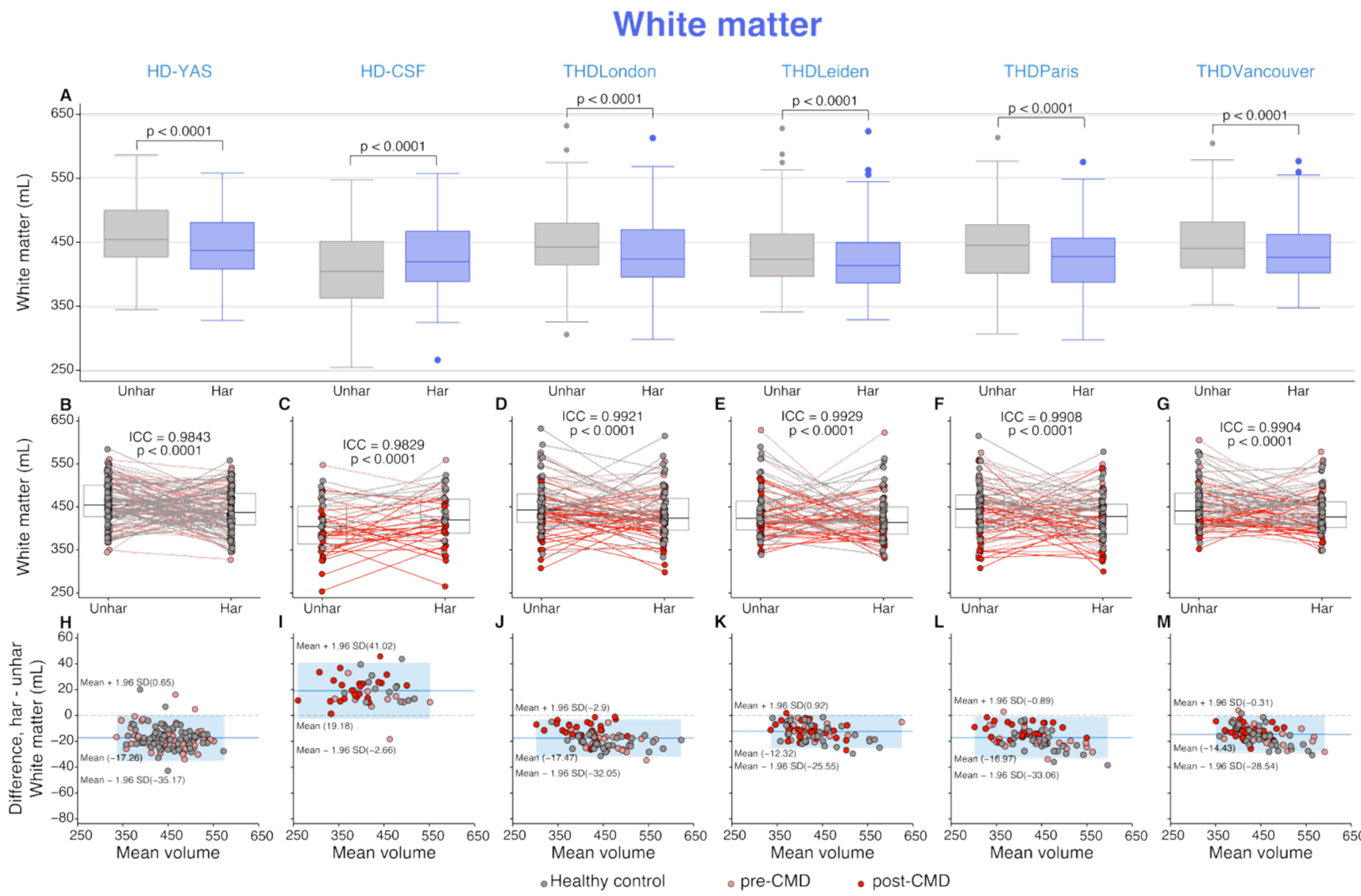

Supplementary Figure 12: Scanner-specific agreement between unharmonized and harmonized white matter volumes. A) Differences between unharmonized and harmonized grey matter volumes for each scanner. P-values extracted from Bonferroni-corrected post-hoc pairwise comparisons of estimated marginal means derived from the mixed-effects model (fixed effects: harmonization × scanner × disease group; random intercept: subject). Boxes show first and third quartiles, the central band shows the median, and the whiskers show data within 1.5 IQR of the median. B-G) Spaghetti plots overlaid on box plots representing the agreement between volumetric measurements before and after harmonization in grey matter for B) HD-YAS, C) HD-CSF, D) TRACK-HD London, E) TRACK-HD Leiden, F) TRACK-HD Paris, and G) TRACK-HD Vancouver. H-M) Bland-Altman plots for agreement between unharmonized and harmonized volumes in grey matter for H) HD-YAS, I) HD-CSF, J) TRACK-HD London, K) TRACK-HD Leiden, L) TRACK-HD Paris, and M) TRACK-HD Vancouver with the 95% limits of agreement represented by the limits of the light blue shaded region; dark blue lines represent the mean difference; dashed line represents y=0. Har, harmonized; ICC, intra-class correlation; post-CMD, pwHD after clinical motor diagnosis; pre-CMD, pwHD before clinical motor diagnosis; THD, TRACK-HD study site; TPM, tissue probability map; Unhar, unharmonized.

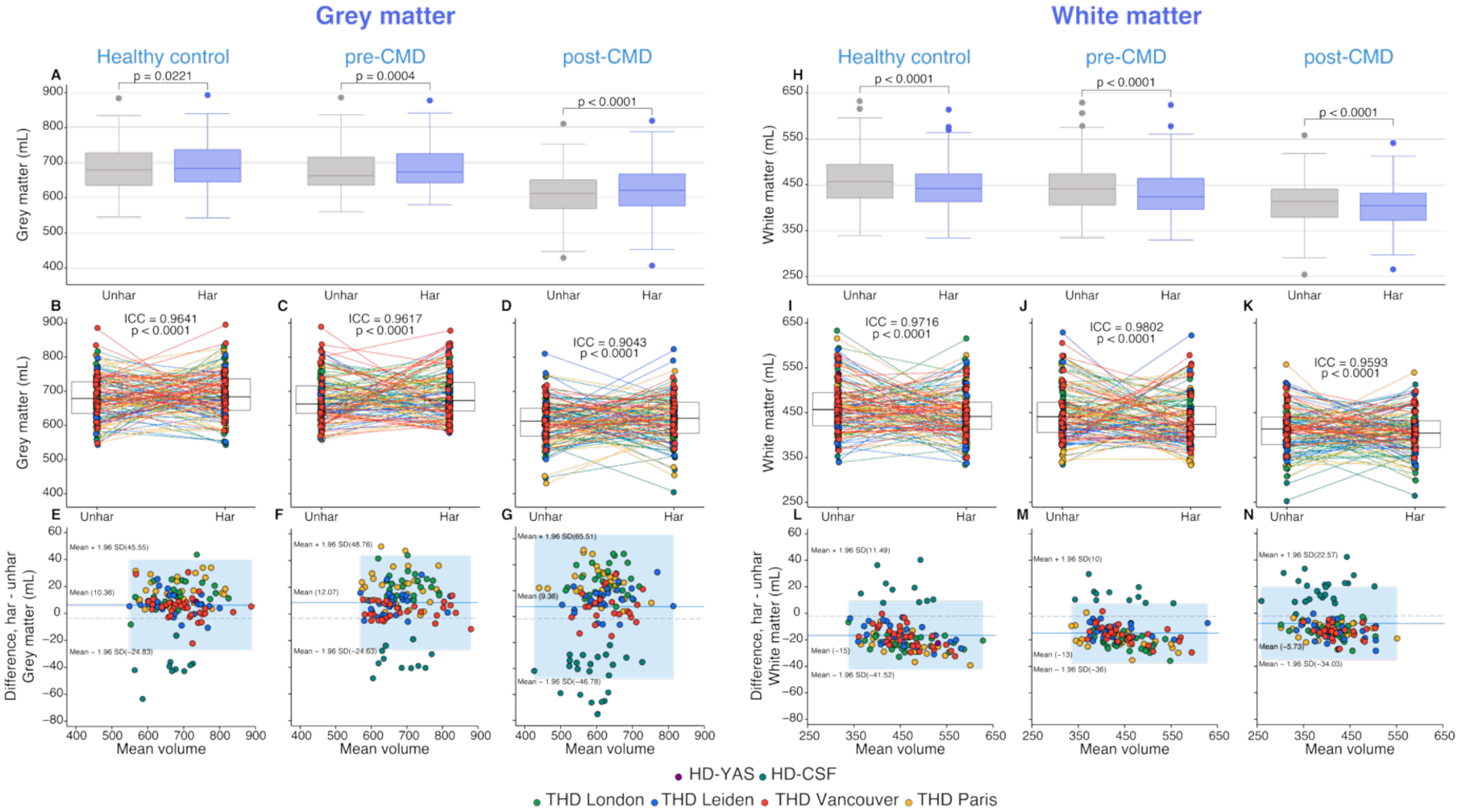

Supplementary Figure 13: Disease-group-specific agreement between unharmonized and harmonized grey and white matter volumes with all QC > 3 excluded (systematic and non-systematic segmentation errors). A,H) Differences between unharmonized and harmonized grey matter A) and white matter H) volumes for each disease group. P-values extracted from Bonferroni-corrected post-hoc pairwise comparisons of estimated marginal means derived from the mixed-effects model (fixed effects: harmonization × scanner × disease group; random intercept: subject). Boxes show first and third quartiles, the central band shows the median, and the whiskers show data within 1.5 IQR of the median. B-D, I-K) Spaghetti plots overlaid on box plots representing the agreement between volumetric measurements before and after harmonization in grey and white matter for B,I) healthy controls, C,J) pre-CMD, and D,K) post-CMD. E-G,L-N) Bland-Altman plots for agreement between unharmonized and harmonized volumes in grey and white matter for E,L) healthy controls, F,M) pre-CMD, and G,N) post-CMD, with the 95% limits of agreement represented by the limits of the light blue shaded region; dark blue lines represent the mean difference; dashed line represents y=0. Har, harmonized; post-CMD, pwHD after clinical motor diagnosis; pre-CMD, pwHD before clinical motor diagnosis; TPMs, tissue probability maps; Unhar, unharmonized.

5 References

Byrne, L. M., Rodrigues, F. B., Johnson, E. B., Wijeratne, P. A., De Vita, E., Alexander, D. C., Palermo, G., Czech, C., Schobel, S., Scahill, R. I., Heslegrave, A., Zetterberg, H., & Wild, E. J. (2018). Evaluation of mutant huntingtin and neurofilament proteins as potential markers in Huntington's disease. *Science Translational Medicine*, *10*(458). https://doi.org/10.1126/scitranslmed.aat7108

Chen, C.-L., Torbati, M. E., Minhas, D. S., Laymon, C. M., Hwang, S. J., Bilgel, M., Crainiceanu, A., Jin, H., Luo, W., Maillard, P., Fletcher, E., Crainiceanu, C. M., DeCarli, C. S., Aizenstein, H. J., & Tudorascu, D. L. (2024). Superpixel-ComBat modeling: A joint approach for harmonization and characterization of inter-scanner variability in T1-weighted images. *Imaging Neuroscience*, *2*, 1–21. https://doi.org/10.1162/IMAG_A_00306

Kieburtz, K. (1996). Unified Huntington's disease rating scale: Reliability and consistency. *Movement Disorders*, *11*(2), 136–142. https://doi.org/10.1002/MDS.870110204

Rodrigues, F. B., Byrne, L. M., Tortelli, R., Johnson, E. B., Wijeratne, P. A., Arridge, M., de Vita, E., Ghazaleh, N., Houghton, R., Furby, H., Alexander, D. C., Tabrizi, S. J., Schobel, S., Scahill, R. I., Heslegrave, A., Zetterberg, H., & Wild, E. J. (2020). Mutant huntingtin and neurofilament light have distinct longitudinal dynamics in Huntington's disease. *Science Translational Medicine*, *12*(574). https://doi.org/10.1126/SCITRANSLMED.ABC2888

Scahill, R. I., Farag, M., Murphy, M. J., Hobbs, N. Z., Leocadi, M., Langley, C., Knights, H., Ciosi, M., Fayer, K., Nakajima, M., Thackeray, O., Gobom, J., Rönnholm, J., Weiner, S., Hassan, Y. R., Ponraj, N. K. P., Estevez-Fraga, C., Parker, C. S., Malone, I. B., … Tabrizi, S. J. (2025). Somatic CAG repeat expansion in blood associates with biomarkers of neurodegeneration in Huntington's disease decades before clinical motor diagnosis. *Nature Medicine 2025*, 1–12. https://doi.org/10.1038/s41591-024-03424-6

Scahill, R. I., Zeun, P., Osborne-Crowley, K., Johnson, E. B., Gregory, S., Parker, C., Lowe, J., Nair, A., O'Callaghan, C., Langley, C., Papoutsi, M., McColgan, P., Estevez-Fraga, C., Fayer, K., Wellington, H., Rodrigues, F. B., Byrne, L. M., Heselgrave, A., Hyare, H., … Tabrizi, S. J. (2020). Biological and clinical characteristics of gene carriers far from predicted onset in the Huntington's disease Young Adult Study (HD-YAS): a cross-

sectional analysis. *The Lancet Neurology*, *19*(6), 502–512. https://doi.org/10.1016/S1474-4422(20)30143-5

Tabrizi, S. J., Langbehn, D. R., Leavitt, B. R., Roos, R. A., Durr, A., Craufurd, D., Kennard, C., Hicks, S. L., Fox, N. C., Scahill, R. I., Borowsky, B., Tobin, A. J., Rosas, H. D., Johnson, H., Reilmann, R., Landwehrmeyer, B., & Stout, J. C. (2009). Biological and clinical manifestations of Huntington's disease in the longitudinal TRACK-HD study: cross-sectional analysis of baseline data. *The Lancet. Neurology*, *8*(9), 791–801. https://doi.org/10.1016/S1474-4422(09)70170-X

Tabrizi, S. J., Reilmann, R., Roos, R. A. C., Durr, A., Leavitt, B., Owen, G., Jones, R., Johnson, H., Craufurd, D., Hicks, S. L., Kennard, C., Landwehrmeyer, B., Stout, J. C., Borowsky, B., Scahill, R. I., Frost, C., & Langbehn, D. R. (2012). Potential endpoints for clinical trials in premanifest and early Huntington's disease in the TRACK-HD study: analysis of 24 month observational data. *The Lancet. Neurology*, *11*(1), 42–53. https://doi.org/10.1016/S1474-4422(11)70263-0

Tabrizi, S. J., Scahill, R. I., Durr, A., Roos, R. A. C., Leavitt, B. R., Jones, R., Landwehrmeyer, G. B., Fox, N. C., Johnson, H., Hicks, S. L., Kennard, C., Craufurd, D., Frost, C., Langbehn, D. R., Reilmann, R., & Stout, J. C. (2011). Biological and clinical changes in premanifest and early stage Huntington's disease in the TRACK-HD study: the 12-month longitudinal analysis. *The Lancet. Neurology*, *10*(1), 31–42. https://doi.org/10.1016/S1474-4422(10)70276-3

Tabrizi, S. J., Scahill, R. I., Owen, G., Durr, A., Leavitt, B. R., Roos, R. A., Borowsky, B., Landwehrmeyer, B., Frost, C., Johnson, H., Craufurd, D., Reilmann, R., Stout, J. C., & Langbehn, D. R. (2013). Predictors of phenotypic progression and disease onset in premanifest and early-stage Huntington's disease in the TRACK-HD study: Analysis of 36-month observational data. *The Lancet Neurology*, *12*(7), 637–649. https://doi.org/10.1016/S1474-4422(13)70088-7
